# Discovery of a small molecule targeting SET-PP2A interaction to overcome BCR-ABLT315I mutation of chronic myeloid leukemia

**DOI:** 10.18632/oncotarget.3665

**Published:** 2015-03-26

**Authors:** Shuzhen Wang, Weiquan Xie, Duowei Wang, Zhigang Peng, Yan Zheng, Nan Liu, Wen Dai, Yang Wang, Zongqiang Wang, Yong Yang, Yijun Chen

**Affiliations:** ^1^ State Key Laboratory of Natural Medicines and Laboratory of Chemical Biology, China Pharmaceutical University, Nanjing, China; ^2^ State Key Laboratory of Natural Medicines and Institute of Pharmaceutical Science, China Pharmaceutical University, Nanjing, China

**Keywords:** chronic myeloid leukemia, BCR-ABL, T315I mutation, SET, protein phosphatase 2A

## Abstract

Despite the great success in using tyrosine kinase inhibitors (TKIs) to treat chronic myeloid leukemia (CML), the frequent development of multi-drug resistance, particularly the T315I mutation of BCR-ABL, remains a challenging issue. Enhancement of protein phosphatase 2A (PP2A) activity by dissociating its endogenous inhibitor SET is an effective approach to combat TKI-based resistance. Here, we report the identification of a novel 2-phenyloxypyrimidine compound TGI1002 to specifically disrupt SET-PP2A interaction. By binding to SET, TGI1002 inhibits SET-PP2A interaction and increases PP2A activity. In addition, knocking-down SET expression decreases tumor cell sensitivity to TGI1002. TGI1002 treatments also markedly increase dephosphorylation of BCR-ABL. Moreover, TGI1002 significantly inhibits tumor growth and prolongs survival of xenografted mice implanted with BaF3-p210^T315I^ cells. These findings demonstrate that TGI1002 is a novel SET inhibitor with important therapeutic potential for the treatment of drug-resistant CML.

## INTRODUCTION

CML is caused by constitutive tyrosine kinase activity of BCR-ABL fusion protein, and great success has been achieved from developing TKIs to treat this deadly disease in clinical settings [1-5]. As the first TKI targeting BCR-ABL kinase, imatinib has been a standard first-line therapy for CML patients [3, 5]. However, approximately one-third of CML patients became clinically resistant to imatinib therapy after an initial response, and the most prevalent mechanism is point mutations within BCR-ABL [3]. To overcome the BCR-ABL mutation-mediated resistance, second-generation TKIs, such as nilotinib, dasatinib and bosutinib, were subsequently developed and have served as salvage therapies for the failures of imatinib treatment. Unfortunately, they were still vulnerable to the frequent gatekeeper mutation of T315I on BCR-ABL [1, 4, 5]. The vexing multi-inhibitor-resistant BCR-ABL^T315I^ mutation spurred the design and development of the third-generation TKIs, among which ponatinib is the only TKI launched to the market [3, 6]. However, FDA halted its clinical uses in 2013 after its accelerated approval for less than a year, due to severe adverse events, such as blood clots and narrowing of blood vessels [6]. Although it was returned to the market later because of the lack of effective alternatives for the treatment of CML patients with BCR-ABL^T315I^ mutation, a black-box warning for arterial thrombotic events was labeled on this drug [6]. Therefore, the frequent and serious multi-TKI resistance has been a major obstacle in the clinical treatment of CML, and novel agents or alternative strategies to overcome BCR-ABL mutations are still badly needed.

**Figure 1:**
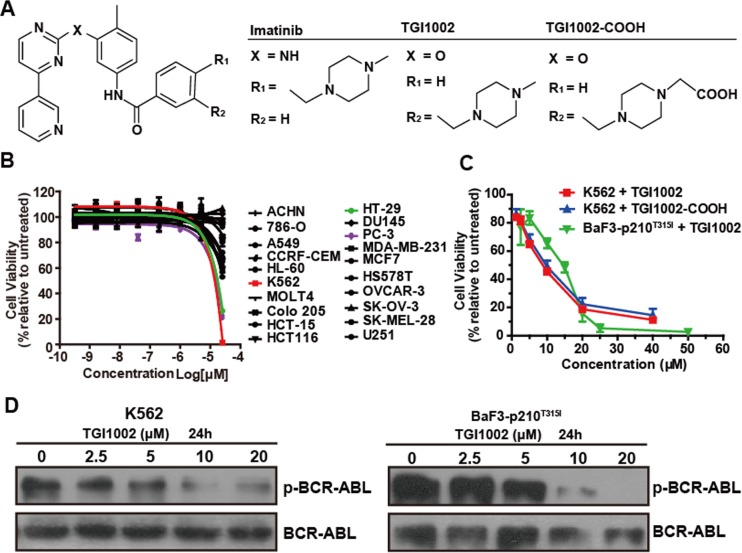
Structure and cytotoxicity of TGI1002 and its analog (**A**) Chemical structure of imatinib, TGI1002 and TGI1002-COOH. (**B**) The cytotoxicity profile of TGI1002 in 20 tumor cell lines. Human tumor cell lines were exposed to TGI1002 for 120 h. Cell viability was determined using CellTiter-Glo Luminescent Cell Viability Assay. (**C**) Dose-response curves for TGI1002 and TGI1002-COOH treatments on K562 and BaF3-p210^T315I^ cells. TGI1002 and its analog TGI1002-COOH inhibited cell proliferation of K562 and BaF3-p210^T315I^ cells in a dose-dependent manner. (**D**) effects of TGI1002 on the expression of BCR-ABL and phosphorylated BCR-ABL in K562 and BaF3-p210^T315I^ cells. Data in (B) and (C) are presented as mean ± s.d. (n= 3).

Previous studies indicated that hydrogen bonding plays a vital role in the binding and inhibition of Abl-1 kinase by imatinib, especially the hydrogen bond between hydroxyl group of Thr315 located at the active site of Abl-1 kinase and 2-amino group at 2-aminophenylpyrimidine core of imatinib [7, 8]. However, the source of proton for the hydrogen bonding between hydroxyl and amino groups was unclear. Because amino group could serve as either proton donor or proton acceptor, hydrogen bonding would be stronger if the amino group only served as a proton acceptor. Therefore, to improve proton accepting capability, we replaced 2-amino group of imatinib by an oxygen atom and synthesized a 2-phenyloxypyrimidine-based compound library to test their inhibitory Effects on kinase activity and tumor cell proliferation. After screening of the library with different substitutions at 2-phenyloxypyrimidine core, we surprisingly found that a novel compound N-(4-methyl-3-((4-(pyridin-3-yl)-pyrimidin-2-yl)oxy)phenyl)-3-((4-methylpiperazin-1-yl)methyl)benzamide hydrochloride (designated as TGI1002, Figure 1A) exhibits unique biological activities, showing anti-proliferation activity to a few tumor cell lines while having no significant inhibition to most tested kinases including Abl-1. Based on its chemical structure and the discrepancy between kinase activity and tumor cell proliferation, the anti-tumor activity of TGI1002 is unlikely to be related to kinase inhibition, implying that a new mechanism of action could be involved in the anti-tumor activity. In an effort to elucidate the mechanism, we used chemical proteomics approach to identify the specific target of TGI1002. Our studies showed that TGI1002 binds specifically to the oncoprotein SET and increases PP2A activity. Furthermore, we demonstrated that TGI1002 is able to overcome multi-drug resistant CML with BCR-ABL^T315I^ mutation through disrupting the interaction between SET and PP2A.

## RESULTS

### Synthesis and evaluation of biological activity of TGI1002

TGI1002 in the form of monohydrochloride was synthesized according to the synthetic route shown in Supplementary Scheme 1. The structure of TGI1002 was characterized by high-resolution mass spectrometry (HRMS), ^1^H NMR, ^13^C NMR and IR (Supplementary Figure S1).

The cytotoxicity of TGI1002 was evaluated in 20 human tumor cell lines using CellTiter-Glo assay. While TGI1002 did not exhibit notable cytotoxicity to most cell lines, cytotoxic effects against K562, PC-3 and HT-29 cells were observed (Figure 1B). Although the IC_50_ value of TGI1002 at 72 h in the most sensitive K562 cells was 11.04 ± 1.98 μM (Figure 1C), approximately 35-fold less potent than imatinib (0.31 ± 0.01 μM), TGI1002 could reduce cell viabilities of TKI-resistant BaF3-p210^T315I^ cells with a comparable IC_50_ value (14.73 ± 2.61 μM) to non-resistant K562 cells (Figure 1C). It should be noted that the increase of TGI1002 sensitivity toward K562 or BaF3-p210^T315I^ BCR-ABL^+^ cells correlated with its ability to induce dephosphorylation of BCR-ABL (Figure 1D).

To examine whether the cytotoxic activity by TGI1002 is related to protein kinase inhibition, a panel of 64 kinases including Tyr and Ser/Thr protein kinases was screened to evaluate the inhibition of TGI1002 on kinase activity using both Kinase-Glo Luminescent Kinase Assay and ADP-Glo Assay. TGI1002 did not inhibit most kinases except showing marginal inhibition on PDGFRα and EphB2 with IC_50_ values of 3.28 μM and 1.27 μM respectively (Supplementary Table S1). Given the fact that PDGFRα and EphB2 are not expressed in K562 and BaF3-p210^T315I^ cells [9, 10] (Supplementary Figure S2), the cytotoxicity by TGI1002 in these cell lines is not related to the inhibition of these kinases, suggesting a mechanism involving other untested kinases or an as yet undefined mechanism.

### Identification of cellular targets of TGI1002

**Figure 2:**
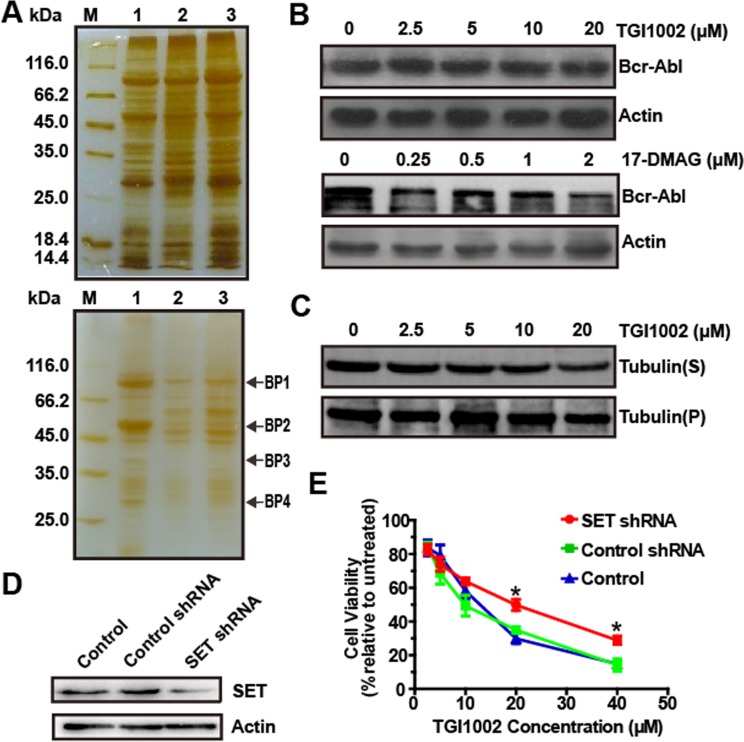
Identification of cellular targets of TGI1002 (**A**) SDS-PAGE for serial affinity chromatography by silver staining. Up panel: blank matrices; down panel: TGI1002-affinity probe. Lane M, protein molecular weight markers. Lane 1-3, Resin-bound proteins from the first, second and third series. Specifically bound proteins are indicated as BP1-BP4. (**B**) Effects of TGI1002 on the expression of BCR-ABL. (**C**) Effects of TGI1002 on microtubules dynamics in K562 cells. Tubulin(S), depolymerized tubulin; Tubulin (P), polymerized tubulin. (**D**) SET protein levels in K562 cells (control) and K562 cells expressing negative control shRNA or SET shRNA. (**E**) TGI1002 sensitivity in K562 cells (control) and K562 cells expressing negative control shRNA or SET shRNA. Data are presented as mean ± s.d. (n = 3).

To elucidate the anti-tumor mechanism and identify potential targets of TGI1002, an affinity probe was prepared as described in Supplementary Scheme 2. We first introduced a carboxylic group onto N-demethylated TGI1002 analogue 10 (Supplementary Figure S3) to yield TGI1002-COOH (Figure 1A and Supplementary Figure S4) and the resulting TGI1002-COOH exhibited a comparable cytotoxicity in K562 cells to the parent compound (Figure 1C), indicating that the additional carboxylic group did not alter its biological activity. TGI1002-COOH was then coupled with EAH-Sepharose 4B through an amide bond and the resulting TGI1002-affinity probe was confirmed by IR spectral comparison (Supplementary Figure S5) and used to incubate with cell lysates of K562 cells. Compared to the non-specific binding proteins with blank matrices (Figure 2A, up panel), four proteins (BP1-BP4) decreased in amount by the affinity probe as shown on SDS-PAGE after serial affinity chromatography [11, 12] (Figure 2A, down panel), indicating that they are binding proteins and potential targets for TGI1002. These four protein bands were subsequently excised from the gel for tryptic digestion and MALDI-TOF MS analyses. MS results revealed that the binding proteins are HSP90AA1, β-tubulin, acetylcholine receptor-associated protein (Rapsyn) and SET protein, respectively (Supplementary Table S2).

Chaperone protein Hsp90 plays an important role in the proper function and stability of a large number of proteins. Hsp90 inhibitors can bind to Hsp90 and specifically inhibit its chaperone function, resulting in the degradation of HSP90-associated proteins, such as BCR-ABL (known as an Hsp90 client protein) in CML cells [13]. Therefore, in the present study, effects of TGI1002 on the expression of BCR-ABL were investigated and compared with a specific Hsp90 inhibitor 17-DMAG. As shown in Figure 2B, the expression level of BCR-ABL in K562 cells was not affected by TGI1002, whereas BCR-ABL was significantly degraded after 17-DMAG treatment, suggesting that HSP90 is not a functional target for TGI1002.

Given that tubulin-binding drugs, such as paclitaxel and *Vinca* alkaloids, act to suppress microtubule dynamics [14], we next examined if TGI1002 plays a role in microtubule-stabilization or microtubule-destabilization in K562 cells. However, the amounts of tubulin in the depolymerized fraction “S” and polymerized fraction “P” were not affected by TGI1002, suggesting that TGI1002 is not involved in the regulation of microtubule dynamics (Figure 2C).

Although 14 tryptic peptide sequences of BP3 matched amino acid sequence of Rapsyn, there was a major discrepancy between the molecular weight of BP3 (~40 kDa) and that of Rapsyn (~47.5 kDa). In addition, Rapsyn has only been known for directing and clustering acetylcholine receptors during cellular differentiation and neuromuscular junction formation [15]. Although the expression of Rapsyn in certain types of nonmuscle cells suggested its additional functions [16], there has been no evidence on its involvement in tumor development. Based on these facts [15, 16], Rapsyn was preliminarily ruled out as a potential target of TGI1002.

To examine whether SET protein mediates the effects of TGI1002, the expression of SET was knocked down in K562 cells and the sensitivity of the cells against TGI1002 was then compared. A K562 cell line with stable knock-down of SET was established using recombinant lentivirus expression vector encoding small hairpin RNA (shRNA) against human SET gene, and suppressed expression of SET was observed by Western blotting (Figure 2D). Subsequent cytotoxicity assays also showed that SET-specific shRNA decreased the sensitivity of K562 cells to TGI1002 treatment (Figure 2E). Together, the data suggest that SET oncoprotein may be the specific target of TGI1002, and we therefore focused our efforts on SET for subsequent investigation.

### Effects on SET binding and PP2A activity by TGI1002

**Figure 3:**
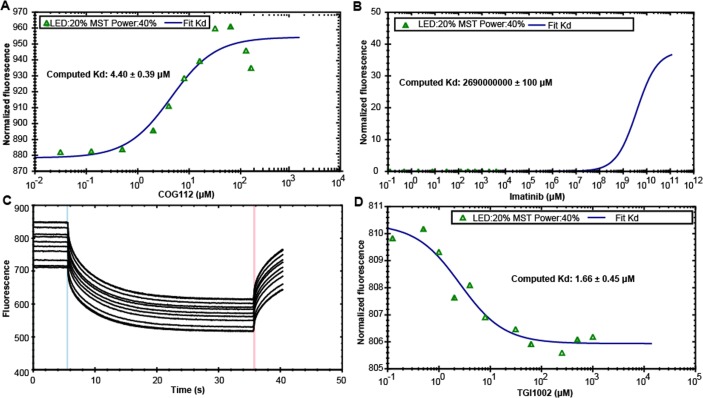
Specific binding of TGI1002 to recombinant human SET oncoprotein measured by MST (**A**) The dissociation constants of COG112 to fluorescently labeled SET. (**B**) The dissociation constants of Imatinib to fluorescently labeled SET. (**C**) MST time traces of 11 concentrations of TGI1002. NT647-labeled SET protein was mixed with increasing TGI1002 concentrations (ranging from 0 to 2 mM). Thermodiffusion is increased with increasing TGI1002 concentrations. (**D**) The dissociation constants of TGI1002 to fluorescently labeled SET. A representative range of data points from two measurements is shown.

To examine whether TGI1002 interacts with SET directly, microscale thermophoresis (MST) was employed in the present study, which enables the quantitative analysis of molecular interactions in solution at microliter scale [17]. Soluble recombinant human SET protein in full length was expressed in *E. coli* [18], purified by Ni-affinity chromatography (Supplementary Figure S6) and labeled with RED fluorescent dye NT-647-NHS. MST was performed by titrating various concentrations of the compounds into NT647-labeled SET protein using COG112, a peptide SET inhibitor [19], as a positive control and imatinib as a negative control. COG112 bound SET protein with a *Kd* value of 4.40±0.39 μΜ (Figure 3A), whereas imatinib did not bind SET to give a meaningful *Kd* value (Figure 3B). TGI1002 bound to SET protein with a *Kd* value of 1.66±0.45 μM (Figure 3C and 3D), exhibiting stronger binding affinity to SET than COG112. These observations provide strong support that TGI1002 interacts directly with SET protein.

**Figure 4:**
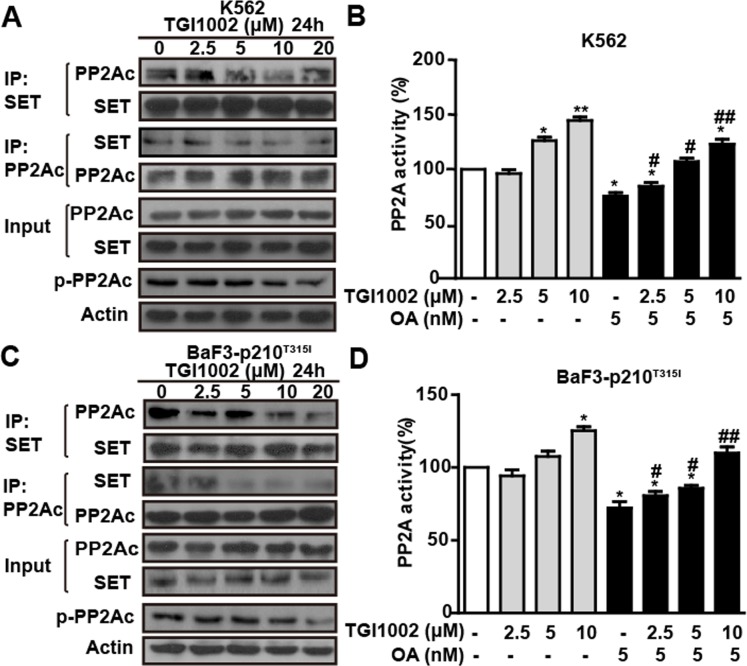
TGI1002 disrupts SET-PP2Ac association and activates PP2A *in vitro* (**A**) TGI1002 inhibited SET-PP2Ac interaction in K562 cells. K562 cells were incubated with TGI1002 for 24 h. SET and PP2Ac protein complexes were immunoprecipitated and analyzed for SET and PP2Ac co-immunoprecipitation. TGI1002 showed inhibition of SET-PP2Ac complex formation. (**B**) TGI1002 treatment activated PP2A in K562 cells. K562 cells were treated with TGI1002 and the activity of PP2A was measured using an immunoprecipitation phosphatase assay. PP2A activity was significantly increased compared to untreated control. (**C**) TGI1002 inhibited SET-PP2Ac interaction in murine BaF3-p210^T315I^ cells. SET and PP2Ac protein complexes were immunoprecipitated and analyzed for SET and PP2Ac co-immunoprecipitation. TGI1002 showed inhibition of SET-PP2Ac complex formation. (**D**) TGI1002 treatment activated PP2A in murine BaF3-p210^T315I^ cells. Data in (B) and (D) are presented as mean ± s.d. (n= 3). *, *P* < 0.05 and **, *P* < 0.01 compared to untreated; #, *P* < 0.05 and ##, *P* < 0.01 compared to OA treatment.

Given that SET is an endogenous inhibitor of the multimeric Ser/Thr protein phosphatase PP2A [18, 19], we next investigated if the binding of TGI1002 with SET protein disrupts the association of SET and PP2A and consequently affect PP2A activity. Co-immunoprecipitation assay showed a trend of decrease on the association between SET and PP2A in K562 cells without significant alteration of SET expression in the presence of TGI1002 (Figure 4A). Meanwhile, there was a tendency on the decrease of catalytic subunit of PP2A (PP2Ac) phosphorylation although lower doses did not show obvious changes (Figure 4A). In addition, PP2A activity, as determined by PP2Ac immunoprecipitation phosphatase assay, was enhanced by 144.67 ± 5.51% after TGI1002-treatment (10 μM, 24 h) in K562 cells compared to untreated cells (Figure 4B). In the presence of specific PP2A inhibitor okadaic acid (OA) [19], PP2A activity decreased significantly compared to control. However, upon TGI1002 treatments, the inhibition of PP2A by OA was reduced in a dose-dependent manner in K562 cells (Figure 4B), further confirming that the increase of PP2A activity was a result of the interaction between TGI1002 and SET. When we treated the drug-resistant BaF3-p210^T315I^ cells, similar to non-resistant K562 cells, TGI1002 disrupted the association of SET with PP2A, down-regulated phosphorylated PP2Ac, increased PP2A activity and antagonized the inhibitory effects of OA on PP2A (Figure 4C and 4D), suggesting that TGI1002 may be active and useful for the treatment of drug-resistant CML with T315I mutation of BCR-ABL.

### *In vivo* confirmation of anti-tumor activity of TGI1002

**Figure 5:**
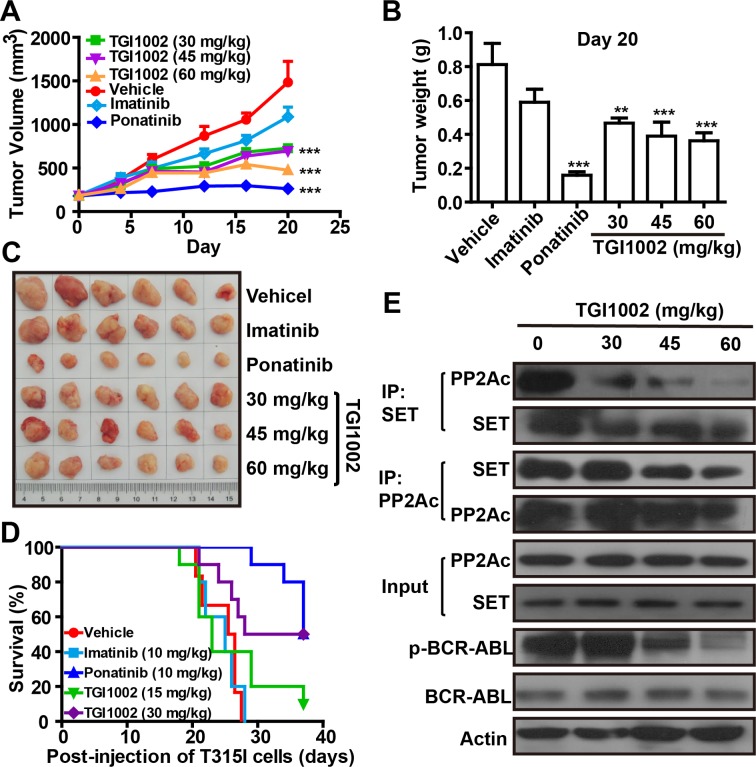
TGI1002 inhibits CML cell growth *in vivo* (**A**) Tumor volumes of BaF3-p210^T315I^ tumor xenografts in BALB/c mice after treatments with 30, 45 and 60 mg/kg TGI1002, 10 mg/kg imatinib or ponatinib, or phosphate saline (vehicle) by intraperitoneal injection. Drug administrations were initiated once tumors reached palpable sizes of 100-200 mm^3^. (**B**) Final tumor mass for TGI1002 treated and untreated BaF3-p210^T315I^ tumors harvested on day 20 after implantation. (**C**) Representative tumors from mice untreated or treated with drugs on day 20. (**D**) Survival curve of BALB/c mice harboring BaF3-p210^T315I^ cells. The mice were injected on day 0 with 10^7^ BaF3-p210^T315I^ cells, and treated beginning on day 5 with imatinib, ponatinib at 10 mg/kg and TGI1002 at 15 and 30 mg/kg once daily by i.p. The survival of TGI1002 (30 mg/kg) and ponatinib-treated mice was significantly longer than vehicle group (*P* = 0.0139 and 0.0001 by log-rank test). (**E**) TGI1002 disrupted SET-PP2Ac association *in vivo*. PP2Ac and SET was immunoprecipitated from homogenized tissues of BaF3-p210^T315I^ cells xenografted tumor treated with saline (vehicle) and TGI1002 (30, 45 and 60 mg/kg). Western blots of SET and PP2Ac indicated the formation of SET-PP2Ac complex in xenografted BaF3-p210^T315I^ tumor tissues treated with vehicle and the disruption of SET-PP2A interaction treated with TGI1002. Data in (A) and (B) are presented as mean ± s.d. (n= 6). **, *P* < 0.01 and ***, *P* < 0.001 compared to vehicle group.

To test *in vivo* anti-tumor activity of TGI1002 and further confirm that TGI1002 directly act on the interaction of SET-PP2A, a xenografted mouse model bearing BaF3-p210^T315I^ was established by implanting tumor cells, and TGI1002 was intraperitoneally administrated to determine anti-tumor efficacy using ponatinib as a positive control. Statistical analyses indicated that tumor growth was significantly inhibited by TGI1002 (Figure 5). The final tumor volume and weight were markedly lower in TGI1002 and ponatinib-treated mice compared to vehicle group, whereas imatinib did not show apparent effects on tumor growth (Figure 5A-C). The inhibition of tumor growth, in terms of tumor volume, of 30, 45 and 60 mg/kg TGI1002 (without loss of body weight) on day 20 post-injection of BaF3-p210^T315I^ cells were 51.07%, 53.15% and 68.01%, respectively. With regard to the survival of tumor-bearing mice, administration of TGI1002 or ponatinib also significantly improved the survival of mice intravenously injected BaF3-p210^T315I^ cells in a dose-dependent manner, while treatment with imatinib had no obvious effects. The median survival of mice xenografted BaF3-p210^T315I^ cells was prolonged to 32.5 days and 37 days by TGI1002 (30 mg/kg) and ponatinib (10 mg/kg) respectively, compared with 25.5 days or 25 days for vehicle or imatinib (10 mg/kg, the effective dose against non-resistant CML) treated mice (Figure 5D).

**Figure 6:**
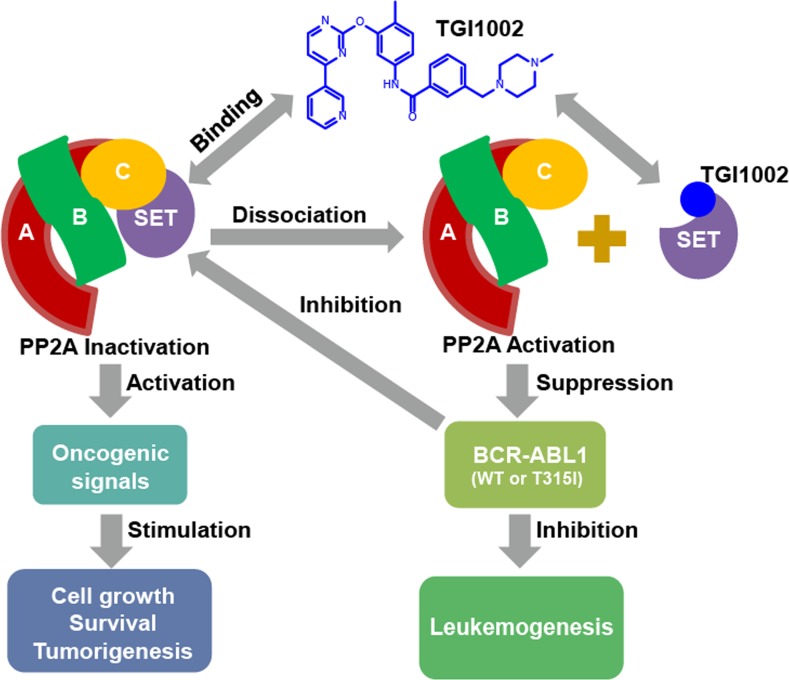
Proposed mechanism of action of TGI1002

To ascertain that the binding of TGI1002 to SET is responsible for the anti-tumor activity of TGI1002 *in vivo*, we analyzed the interaction of SET with PP2A in tumor tissues from the xenografted mice. As anticipated, the disrupting effects of TGI1002 were also observed in tumor tissues. Further analysis of other related proteins showed that the levels of SET and BCR-ABL were not affected by TGI1002, whereas the levels of phosphorylated BCR-ABL (active form of BCR-ABL) were markedly reduced upon TGI1002 treatment (Figure 5E). Collectively, these results strongly support that the disruption of SET-PP2A interaction by TGI1002 results in the decrease of tumor growth and extension of survival of tumor-bearing mice (Figure 6).

## DISCUSSION

Given the decisive role of BCR-ABL in the development of CML, TKI-based therapy has been regarded as the gold standard for the treatment of CML since the excellent clinical performance of the first TKI, imatinib. The magic bullet of imatinib did revolutionize CML therapy and bring substantial benefits to CML patients. However, it was also the initiator of BCR-ABL mutations to acquire drug resistance. Since then, numerous efforts have been put on the road to battle various BCR-ABL mutations, and second and third generations of TKIs have been developed over the past decade [3]. Unfortunately, the pace of drug development has not been able to keep up with the emergence of drug resistance. Recently, T315I-inclusive compound mutations conferring ponatinib (the only currently approved pan-BCR-ABL inhibitor) resistance was reported [20, 21]. This serious situation highlights the need to develop new therapeutic strategies to offer more options for multi-drug resistant CML patients.

Imatinib contains a 2-aminophenylprimidine core structure, in which hydrogen bondings are critical for competitive binding with ATP binding packet to inactivate Abl-1 kinase activity [7, 8]. Among the hydrogen bonds, the one between 2-amino group at 2-aminophenylpyrimidine core of imatinib and the gatekeeper residue Thr315 of Abl-1 kinase is particularly important according to the small molecule-protein complex structure [7, 8]. In the present study, we designed and synthesized a 2-phenyloxypyrimidine-based library aiming to increase proton accepting capability and consequent stronger inhibition on Abl-1 kinase. To our surprise, we found that compound TGI1002 shows moderate cytotoxicity only to a few cell lines and the cytotoxicity is not related to kinase inhibition, suggesting that a different mechanism from imatinib underlies its anti-tumor activity.

Affinity-based chemical proteomics has been a powerful tool to identify the molecular targets of bioactive small molecules [11, 12, 22]. In the present study, we employed this approach to examine if TGI1002 can bind to a specific protein in K562 cells. After three rounds of serial affinity chromatography, four proteins were captured by the TGI1002-affinity probe and subsequently identified by MALDI-TOF-MS analyses. After preliminarily excluding the involvement of other proteins, SET oncoprotein was confirmed to be the specific-binding protein responsible for the anti-tumor activity of TGI1002 with following evidence: (1) shRNA-mediated knock-down of SET expression significantly reduced the sensitivity of K562 cells to TGI1002; (2) TGI1002 could directly bind recombinant human SET protein with high affinity determined by MST; (3) the disruption of SET-PP2A interaction by TGI1002 was observed from co-imminoprecipitaton in both tumor cell lines and tissues; and (4) a dose-dependent efficacy by TGI1002 in xenografted mice implanted with drug-resistant BaF3-p210^T315I^ cells was confirmed from both tumor growth and survival.

SET oncoprotein is a potent and specific endogenous inhibitor of tumor suppressor PP2A in cytoplasm, its over-expression and inactivation of PP2A activity was recently recognized as a common event in leukemogenesis [23-27]. Hence, increase of cytosolic PP2A activity through dissociating SET by PP2A-activating drugs or SET-binding peptides has been indicated as a promising approach to overcome TKI-based drug resistance [28, 29]. Recent studies have resulted in the identification of sphingosine analogue FTY720 (fingolimod, Gilenya; Novartis) and SET binding peptides, such as COG112 and OP449, as potential therapeutic agents for the treatment of leukemogenesis [29-32].

In this study, the ability of TGI1002 to dissociate SET from PP2A and to increase PP2A activity was observed both *in vitro* and *in vivo*. Decrease of PP2A phosphorylation was previously regarded as a major event to increase PP2A activity [26, 27]. The present study did show a tendency of decrease in PP2A phosphorylation upon TGI1002 treatments at high doses. However, no significant changes were observed at low concentrations of TGI1002. Therefore, we reason that the increase of PP2A activity by TGI1002 could be a synergetic effect of SET inhibition and PP2A dephosphorylation. Because the efficacy of peptide SET inhibitor OP449 was only confirmed in mice xenografted with drug-sensitive human acute myeloid leukemia HL-60 cells [29], ponatinib, the only therapeutic agent for T315I mutation of CML patients, was chosen as a positive control for the comparison of *in vivo* efficacy. Although the *in vivo* anti-tumor efficacy of TGI1002 was less potent than the typical TKI ponatinib, potential clinical efficacy of TGI1002 may not be necessarily lower under complex disease conditions due to the mechanistic difference. Meanwhile, it would be of interest to evaluate the synergetic effects by combining TGI1002 with other therapeutic agents possessing different mechanisms, and such combinational therapies are currently under investigation.

In summary, the present study provided a novel compound and chemotype to combat multi-drug resistant CML, particularly the T315I mutation. Compound TGI1002 containing 2-phenoxypyrimidine structure exerted its anti-leukemic activity by directly interacting with SET oncoprotein to disrupt the association of SET and PP2A, resulting in the activation of PP2A and the suppression of BCR-ABL activity. To address the important unmet medical needs, the novel compound, new chemotype and the mechanism of action revealed in the present study provide a potentially new treatment option for the patients with Philadelphia choromosome-positive leukemias.

## MATERIALs AND METHODS

### Materials

Imatinib mesylate and ponatinib hydrochloride were obtained from NCE Biomedical Co., Ltd. ApoE-mimetic peptide COG112 (acetyl-LRVRLASHLRKLRKRLL-amide) was synthesized by ChinaPeptides Co., Ltd and purified to > 95% purity. Antibodies to BCR-ABL (b2a2 Junction Specific) (#3908), p-Abl (Tyr245) (#2861) and PP2Ac (#2038) were from CST; antibodies to p-PP2Ac (Y307) (#sc-12615-R), EphB2 (#sc-1763), PDGFRα (#sc-338), β-actin (#sc-47778) and β-tubulin (#sc-9104) were from Santa Cruz; antibodies to SET (#ab1183) was from Abcam. 17-DMAG was acquired from Selleck Chemicals. Okadaic acid (OA) and other reagents and solvents were analytical grade and from Sigma-Aldrich unless noted otherwise.

### Cell culture

Tumor cell lines of ACHN, 786-O, A549, CCRF-CEM, HL-60, K562, MOLT4, Colo 205, HCT-15, HCT116, HT-29, DU145, PC-3, MDA-MB-231, MCF7, HS578T, OVCAR-3, SK-OV-3, SK-MEL and U251 were obtained in February 2012 from American Type Culture Collection (ATCC) and authenticated by microsatellite genotyping. A549 and PC-3 cells were grown in F12K medium; 786-O, DU145, MDA-MB-231, MCF7, HS578T, SK-MEL-28 and U251 cells were grown in DMEM medium; ACHN, CCRF-CEM, HL-60, K562, MOLT4, Colo 205, HCT-15 and OVCAR-3 cells were grown in RPMI 1640 medium; HT-29, HCT116 and SK-OV-3 cells were grown in McCoy's 5A medium. BaF3 cell lines harboring either wild type (p210) or T315I mutation of BCR-ABL kinase (p210^T315I^) were generous gifts from Prof. Xiaoguang Chen (Chinese Academy of Medical Sciences & Peking Union Medical College, Beijing, China) and cultured in RPMI 1640 medium. All media contained 10% fetal bovine serum (FBS) in the presence of 100 units/mL penicillin and 0.1 mg/mL streptomycin. Cells were incubated at 37 °C with 95% air and 5% CO_2_.

### Synthesis of TGI1002 and TGI1002-affinity probe

TGI1002 was synthesized according to the synthetic route (Supplementary Scheme 1). The structure of TGI1002 was determined by HRMS, ^1^H NMR, ^13^C NMR and IR. TGI1002-affinity probe was then synthesized from TGI1002 (Supplementary Scheme 2). First, an N-demethyl-derivative of TGI1002 (10) was synthesized based on Supplementary Scheme 1 with slight change, in which 1-methyl-piperazine was replaced by piperazine. Then, TGI1002-COOH was synthesized from 10 based on a previous report [34]. The structures of 10 and TGI1002-COOH were confirmed by HRMS, ^1^H NMR, ^13^C NMR and IR. TGI1002-affinity probe was prepared by coupling TGI1002-COOH with EAH-sepharose 4B with the same conditions as described previously [11, 12]. Briefly, 0.5 mL EAH-sepharose 4B (GE Healthcare Biosciences AB) was successively washed with deionized water (pH 4.5), 0.5 M NaCl and PBS (pH 7.4), and suspended in PBS (pH 7.4). TGI1002-COOH (2.5 mg, 4.4 μmol) dissolved in 0.5 mL deionized water (pH 4.5) and 0.1 M 1-(3-dimethylaminopropyl)-3-ethylcarbodiimide hydrochloride (EDC) were added to the resin suspension. The mixture was stirred overnight at room temperature and dried. The resulting matrices were washed thoroughly with methanol and water, characterized by IR spectroscopy and kept in an aqueous solution containing 20% ethanol until used in binding experiments. The coupling rate of TGI1002-COOH to EAH-sepharose was 32.1% by determining the residual TGI1002-COOH using HPLC.

### Cytotoxicity assay

Twenty types of tumor cell lines were seeded at 4000 cells per well in 96-well plates. TGI1002 dissolved in phosphate saline was added to cells at final concentrations ranging from 0.5 nM to 100 μM for 120 h. Cell viability was measured with the CellTiter-Glo Luminescent Cell Viability Assay (Promega) according to the manufacturer's instructions. Luminescence was measured using an EnVision Multilabel Plate Reader (Perkin-Elmer). The cytotoxicity of TGI1002 was expressed as the percentage of luminescence relative to that of untreated cells.

### *In vitro* kinase assay

All kinase reactions were carried out in 384-well plates. After addition of the compounds, kinase and ATP-substrate were added to the assay plates successively, the assay plates were centrifuged at 1,000 x *g* for 1 min and incubated for 1 h at 30 °C. Then, Kinase-Glo Plus or ADP-Glo reagent was added to each well. In Kinase-Glo assay, after addition of the reagent for 20 min, the luminescence was measured on an EnVision Multilabel Plate Reader (Perkin-Elmer). In ADP-Glo assay, after addition of the reagent for 40 min, Kinase Detection Reagent was added and incubated for another 30 min at 27 °C, and then the luminescence was measured. Kinase inhibition rates were calculated per manufacturer's instructions.

### Capture and identification of specific binding proteins

According to previously reported method of serial affinity chromatography^10,11^, lysates of K562 cells (200 μL) were stirred gently with TGI1002-affinity probe (60 μL) or blank matrices (EAH sepharose 4B) at 4 °C for approximately 40 min and then precipitated by centrifugation at 8,000 x *g* for 3 min. The supernatants were mixed with 60 μL of TGI1002-affinity probe or blank matrices at 4 °C, and incubated for approximately 40 min. The resulting resins were successively washed for three series with 0.6 ml of lysate buffer containing 20 mM Tris-HCl (pH 7.0), 150 mM NaCl, 1 mM EDTA, 1 mM MgCl and 0.5% NP-40 supplemented with a cocktail of protease and phosphatase inhibitors (Roche). The mixtures were then resuspended in 40 μL of SDS loading buffer, boiled at 100 °C for 5 min, and then centrifuged for 3 min. The supernatants were subjected to SDS-PAGE. After silver staining of the gels, the protein bands were comparatively analyzed between the affinity probe and blank matrices to identify specific binding proteins. Individual protein band on the SDS-PAGE gel was excised and digested with trypsin. Tryptic peptides were analyzed on an Ultraflex II MALDI-TOF/TOF instrument (Bruker Daltonics, Bremen, Germany) in positive-ionization mode over the m/z range of 700 to 4000 at resolution of 15,000 to 20,000. Protein sequence identification was performed with the peptide mass fingerprint using the Mascot Search engine (version 2.4) as previously reported [35].

### Microtubule assembly assay

Separation of insoluble polymerized tubulin from soluble tubulin dimers and analysis of the effects of TGI1002 on tubulin polymerization were performed as described previously [36]. In brief, K562 cells at a density of 1 × 10^6^/100 mm^2^ dishes were treated with indicated concentrations of TGI1002 at 24 h. Cells were washed with PBS for three times before adding polymerization lysate buffer containing 20 mM Tris-HCl (pH6.8), 1 mM MgCl_2_, 2 mM EGTA, 20 g/mL aprotinin, 20 g/mL leupeptin, 1 mM phenylmethylsulfonyl fluoride, 1 mM orthovanadate and 0.5% Nonidet. After centrifugation at 15,000 x g for 10 min at 4 °C, the pellets were resuspended in SDS-PAGE loading buffer and dissolved by heating at 95 °C for 10 min. Beta-tubulin was then Western blotted from both pellets (containing polymerized microtubules) and supernatants (containing depolymerized microtubules).

### Stable transfection and selection of K562 cell line with SET-knockdown

Lentivirus-encoded shRNA against human SET (GenBank No.: NM_003011) and a noncoding gene (negative control) were prepared by Genechem and titered to 2 × 10^8^ (TU/mL). K562 cells (1 × 10^4^ cells/well) were seeded in 96-well plates overnight before transfection. The virus (10 μL) was mixed with 90 μL complete medium containing polybrene (5 μg/mL) and added to cells and incubated for 12 h at 37 °C. Then, the cells were incubated in fresh complete RPMI 1640 medium for 72 h, followed by incubation in complete medium containing puromycin (5 μg/mL). After 2 weeks of selection, individual clones were isolated, passaged and harvested for subsequent studies. Identification of stable SET-knockdown K562 cell line was performed by Western blotting. Effects of TGI1002 on cell viability of SET-knockdown K562 cells were studied as described above.

### Soluble expression and purification of human SET protein in *E. coli*

Genes encoding the full length of human SET (GenBank No.: AAA60318.1) was synthesized by Shanghai Generay Biotech Co., Ltd and subcloned into pET-21a expression vector. The expression and purification of recombinant human SET protein was carried out as described previously [18]. The protein was purified to homogeneity on SDS-PAGE and concentrated with a centrifugal filter device (Millipore). Protein concentrations were quantified by BCA assay (KeyGen).

### Fluorescence microscale thermophoresis (MST)

Purified SET protein was labeled with dye NT647 using a Monolith NT^TM^ Protein Labeling Kit (NanoTemper Technologies). Serial dilutions of TGI1002, COG112 or imatinib (up to 2 mM) were mixed with 15 μM of NT647-labeled SET protein, incubated for 10 min at room temperature in 20 mM PBS (pH 8.0) and loaded into the standard glass capillaries (Monolith NT Capillaries, NanoTemper). Thermophoresis analysis was performed during 30 s on a NanoTemper Monolith NT.115 instrument (20% LED, 40% MST power) at 25 °C. The MST curves were fitted using NT Analysis software (NanoTemper Technologies) to obtain *Kd* values for binding between SET and TGI1002, COG112 or imatinib, respectively.

### Western blotting and immunoprecipitation

Protein samples (40 μg) were resolved on SDS-PAGE gels, transferred onto PVDF membranes (Millipore) and incubated overnight with primary antibodies to β-tubulin, BCR-ABL, p-BCR-ABL, SET, PP2Ac and p-PP2Ac. The membranes were incubated with horseradish peroxidase (HRP)-conjugated secondary antibody for 1 h, and the bands were visualized using an ECL detection kit following the manufacturer's instructions. For immunoprecipitation, cells were lysed in 20 mM Tris-HCl (pH 7.0), 150 mM NaCl, 1 mM EDTA, 1 mM MgCl and 0.5% NP-40 supplemented with a cocktail of protease and phosphatase inhibitors (Roche). Lysates (200 μg) were incubated with primary antibodies (1 μg) against SET or PP2Ac at 4 °C for 10 h, protein A/G plus agarose beads were then added for immunoprecipitation overnight at 4 °C. After wash, the immunoprecipitated proteins were separated by SDS-PAGE and analyzed by Western blotting.

### PP2A activity assay

PP2A assay from whole cell lysates was carried out using commercial PP2Ac immunoprecipitation phosphatase assay kit (Millipore). Briefly, 200 μg protein lysates prepared as described above, 4 μg PP2Ac-specific antibody, and 40 μL protein A-agarose were added to TBS buffer (20 mM Tris-HCl, 150 mM NaCl, and 0.1% Tween 20) to a final volume of 500 μL and immunoprecipitation was carried out at 4 °C for 2 h. The immunoprecipitates were used in the phosphatase reaction following the manufacturer's protocol.

### *In vivo* anti-tumor efficacy

BABL/c male mice from SLRC Experimental Animal Center were bred under pathogen-free conditions and were used for experiments at 6-8 weeks of age. Animal experiments were performed according to institutional and national guidelines. BaF3-p210^T315I^ cells were implanted subcutaneously into the right flank of nude mice (200 μL of a 1 × 10^7^cells suspension in serum free medium). When tumors became large enough for caliper measurement, tumor volumes were calculated as (length × width^2^) × 0.5. Once tumors reached 100-200 mm^3^, mice were randomly assigned to 6 groups with approximate equalization of tumor volumes among different groups. TGI1002 (30, 45 and 60 mg/kg), imatinib (10 mg/kg), ponatinib (10 mg/kg), or vehicle (phosphate saline) was administered daily by intraperitoneal injection (i.p.). At the end of the experiments, tumors were dissected and photographed, and wet weights of each tumor were measured.

In survival study, BaF3-p210^T315I^ cells were injected into the tail vein of BALB/c mice (100 μL of a 1 × 10^7^ cells suspension in serum free medium). Five days later, mice were treated once daily by i.p. with vehicle, TGI1002 (15, 30 mg/kg), imatinib (10 mg/kg) and ponatinib (10 mg/kg). Moribund animals were sacrificed as per institutional and national guidelines. Survival data were analyzed by the Kaplan Meier method and compared using the log-rank test.

### Statistical analysis

All statistical analyses were conducted using GraphPad Prism version 6.01 for Windows. Data are expressed as mean ± s.d. of three independent experiments. The significance of the data was determined by one-way ANOVA analysis unless otherwise noted. *P* value < 0.05 was considered statistically significant.

## SUPPLEMENTARY MATERIAL FIGURES AND TABLES


