# Supplementary material for: Discovery of a small molecule targeting SET-PP2A interaction to overcome BCR-ABLT315I mutation of chronic myeloid leukemia
Source: Oncotarget. 2015 Mar 26;6(14):12128–40. doi: 10.18632/oncotarget.3665 (PMC4494927; doi:10.18632/oncotarget.3665)
Supplement: Supplementary file 1 [file oncotarget-06-12128-s001.pdf]

Discovery of a small molecule targeting SET-PP2A interaction to overcome BCR-ABL^{T315I} mutation of chronic myeloid leukemia

Supplementary Material

Table of contents

Materials & Instrumentation.. 2

Synthesis... 3

Reference.. 10

Supplementary Scheme 1: Synthetic route of TGI1002................................ 11

Supplementary Scheme 2: Synthesis of TGI1002 derivative as an affinity probe... 12

Supplementary Figure S1: HRMS, ^{1}H and ^{13}C NMR and IR analyses of TGI1002.. 13

Supplementary Figure S2: Western blots of PDGFRα and EphB2 expression in K562 and BaF3-p210^{T315I}cells.. 16

Supplementary Figure S3: HRMS, ^{1}H and ^{13}C NMR and IR analyses of **10**......... 17

Supplementary Figure S4: HRMS, ^{1}H and ^{13}C NMR and IR analyses of TGI1002-COOH... 20

Supplementary Figure S5: IR spectral analysis of TGI1002-affinity probe......... 23

Supplementary Figure S6: SDS-PAGE of purified SET protein...................... 25

Supplementary Table S1: *In vitro* profile on kinase inhibition of TGI1002.......... 26

Supplementary Table S2: MALDI-TOF-TOF analysis of TGI1002 binding proteins.. 27

Materials & Instrumentation

The starting materials, other reagents and solvents for chemical synthesis were acquired from Sigma-Aldrich unless otherwise noted. Melting points were determined using YRT-3 melting point detector. High resolution mass spectra (HRMS) were obtained on 6200 series TOF/6500 series spectrometer (Agilent Technologies). ^{1}H NMR and ^{13}C NMR spectra were recorded on Bruker AV300 NMR spectrometer. IR spectra were measured by Bruker Tensor 27 FT-IR spectrometer.

Synthesis

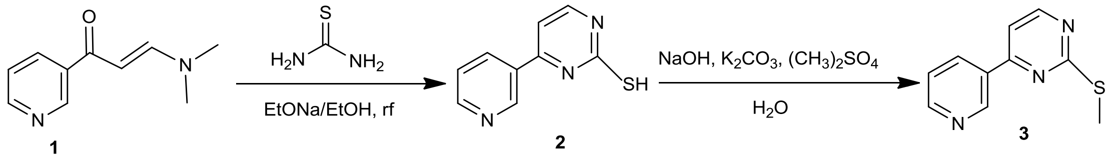

Preparation of 2-(methylthio)-4-(3-pyridinyl)pyrimidine (3). Sodium ethoxide (13.91 g, 0.2 mol) was dissolved in 150 mL anhydrous ethanol, then 15.55 g thiourea (0.2 mol) and 3-dimethylamino-1-(3-pyridin-2-yl)prop-2-en-one (**1**) (30 g, 0.17 mol) was added and heated to reflux for 2-3 h. Water (25 mL) was added into the solution. The mixture was then acidified with acetic acid to pH≈5 and kept for 30 min under 0 ℃ to give yellow precipitate. After removing the solvent, the precipitate was washed with water to afford 2-methyl-4-(pyridin-2-yl)pyrimidine hydrosulfide (**2**). The above obtained compound **2,** K_2CO_3 (23.46 g, 0.17 mol) and NaOH (4.76 g, 0.12 mol) were dissolved in 200 mL water. Dimethyl sulfate (25.79 g, 0.2 mol) was added dropwise with stirring at 0-5 ℃ within 30 min, after which 60 mL water was added to the reaction solution. The precipitate was washed with water and dried to give **3** as a yellow powder (23.7 g, purity>98%, yield 68.5%). Mp: 106-108 ℃; MS-ESI$^+$ m/z, 204 (MH$^+$); ^{1}H NMR (300 MHz, $CDCl_3$): δ 9.28 (d, 1H, J=2.3 Hz), 8.74 (dd, 1H, J=1.5, 4.8 Hz), 8.61 (d, 1H, J=5.3 Hz), 8.42 (m, 1H), 7.45 (m, 1H), 7.41 (d, 1H, J=5.3 Hz), 2.65 (s, 3H).

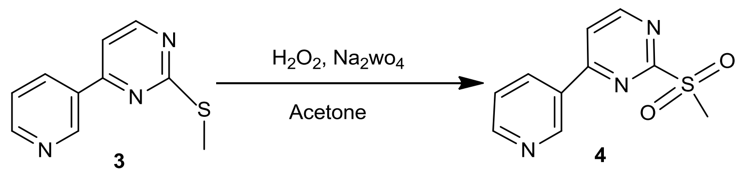

Preparation of 2-(methylsulfonyl)-4-(pyridin-3-yl)pyrimidine (4). Compound **3** (9.81 g, 45 mmol) was dissolved in 45 mL acetone and heated to reflux for 30 min, and Na_2WO_4 (0.91 g) was added. After stirring for 10 min, H_2O_2 (12.34 g, 89 mmol) was added dropwise within 30 min. The solution was heated to reflux for 3-5 h, after which insoluble solid was formed. $Na_2S_2O_3$ (4.5 g) dissolved in 100 mL was added to the mixture with stirring, and kept for 30 min at 0-5 ℃. After removing the solvent, the residue was dried at 60 ℃ to afford **4** (8.5 g, purity > 95%, yield 80%). Mp: 151-153 ℃; MS-ESI$^+$ m/z, 236 (MH$^+$); ^{1}H NMR (300 MHz, $CDCl_3$): δ 9.34 (dd, 1H, J=0.8, 2.4 Hz), 9.01 (d, 1H, J=5.3 Hz), 8.82 (dd, 1H, J=1.6, 4.7 Hz), 8.54 (dd, 1H, J=1.6, 2.4 Hz), 7.99 (d, 1H, J=5.3 Hz), 7.52 (m, 1H), 3.45 (s, 3H).

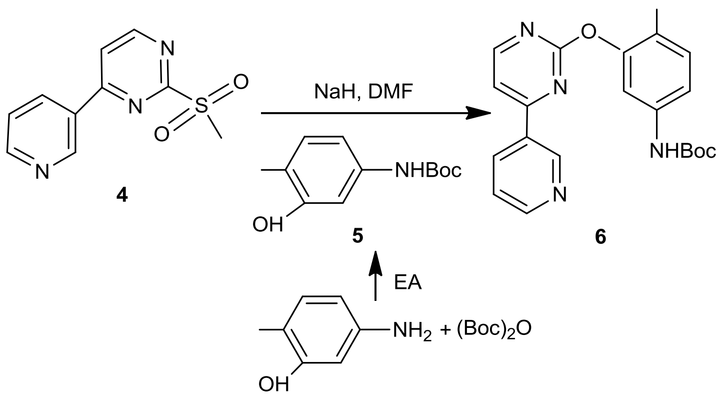

Preparation of tert-butyl (4-methyl-3-((4-(pyridin-3-yl)pyrimidin-2-yl) -oxy)phenyl)-carbamate (6). 5-Amino-2-methylphenol (25 g, 0.203 mol) was dissolved in 400 mL ethyl acetate (EA), and $(Boc)_2O$ (47 g, 0.203 mol) was then added with stirring. The solution was stirred for 24 h at room temperature (RT), washed with 10% NaOH and water, and dried and concentrated. After isolating by column chromatography (ethyl acetate/ethane = 1/5), tert-butyl (3-hydroxy-4-methylphenyl)carbamate (**5**) was obtained as a white solid (43 g, yield 95%). Compound **4** (20.75 g, 0.88 mol) and **5** (19.62 g, 0.88 mol) were dissolved in 100 mL N,N-dimethylformamide (DMF), and 60% NaH (6.02 g, 0.25 mol) was then added with stirring at 0 ℃. The reaction was carried out at 40~50 ℃ for 0.5~1 h. The pH of the reaction solution was then adjusted with 300 mL 3N HCl to 5~6, followed by adding 200 mL ice-water, and stirred for 30 min at RT. After removing the solvent, the light yellow solid was dried to give compound **6** (32.17 g, purity > 95%, yield 80%). Mp:161-162 ℃; MS-ESI$^+$ m/z 379 (MH^+); ^{1}H NMR (300 MHz, $CDCl_3$): δ 9.22 (dd, 1H, J=0.8, 2.3 Hz), 8.72 (dd, 1H, J=1.5, 4.6 Hz), 8.61 (d, 1H, J=5.1 Hz), 8.36 (m, 1H), 7.46 (d, 1H, J=5.1 Hz), 7.42 (m, 1H), 7.37 (br, 1H), 7.19 (d, 1H, J-8.3 Hz), 7.08 (m, 1H), 6.58 (br, 1H), 2.15 (S, 3H), 1.49 (S, 9H).

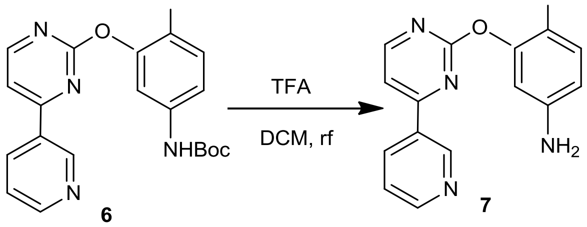

6 7

Preparation of 4-methyl-3-((4-(pyridin-3-yl)pyrimidin-2-yl)oxy)aniline (7). Compound **6** (32.17 g, 85 mmol) was dissolved in 130 mL dichloromethane (DCM), in which 100 g trifluoroacetate was added at 0 ℃. The solution was heated to reflux for 2-3 h, and then pH was adjusted to 8-9 with 10% NaOH. The organic phase was washed with water, and DCM was removed by evaporation. The residue was treated with 50 mL ethanol for 1 h, filtered and dried to afford yellow solid **7** (23.7 g, purity > 98%, yield 95%). Mp: 155-157 ℃; MS-ESI$^+$ m/z 279 (MH^+); ^1H NMR (300 MHz, $CDCl_3$): δ 9.23 (d, 1H, J=2.3 Hz), 8.73 (dd, 1H, J=1.5, 4.7 Hz), 8.62 (d, 1H, J=5.3 Hz), 8.37 (m, 1H), 7.46 (d, 1H, J=5.3 Hz), 7.43 (m, 1H), 7.08 (d, 1H, J=8.0 Hz), 6.52-6.56 (m, 2H), 2.07 (S, 3H).

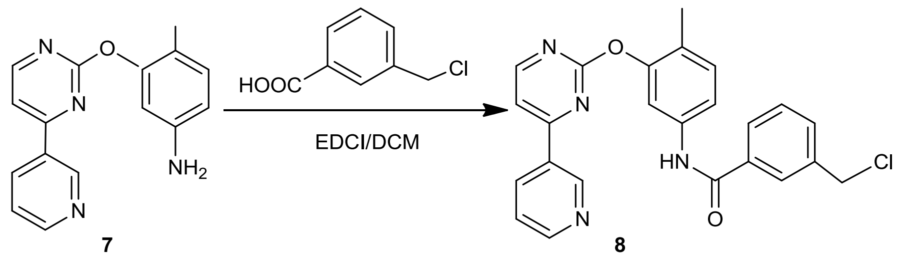

7 8

Preparation of 3-(chloromethyl)-N-(4-methyl-3-((4-(pyridin-3-yl)pyrimidin -2-yl)-oxy)phenyl)-benzamide (8). 3-(Chloromethyl)benzoic acid (12.6 g, 74 mmol) and EDCl (17 g, 89 mmol) were dissolved in 110 mL DCM, followed by adding 13.7 g compound **7** at 10 ℃, and heated to reflux for 1-2 h. The solution was washed successively with 250 mL water and $NaHCO_3$. After removing the organic phase, the yellow solid **8** was obtained (12.5 g, purity > 95%, yield 59%).

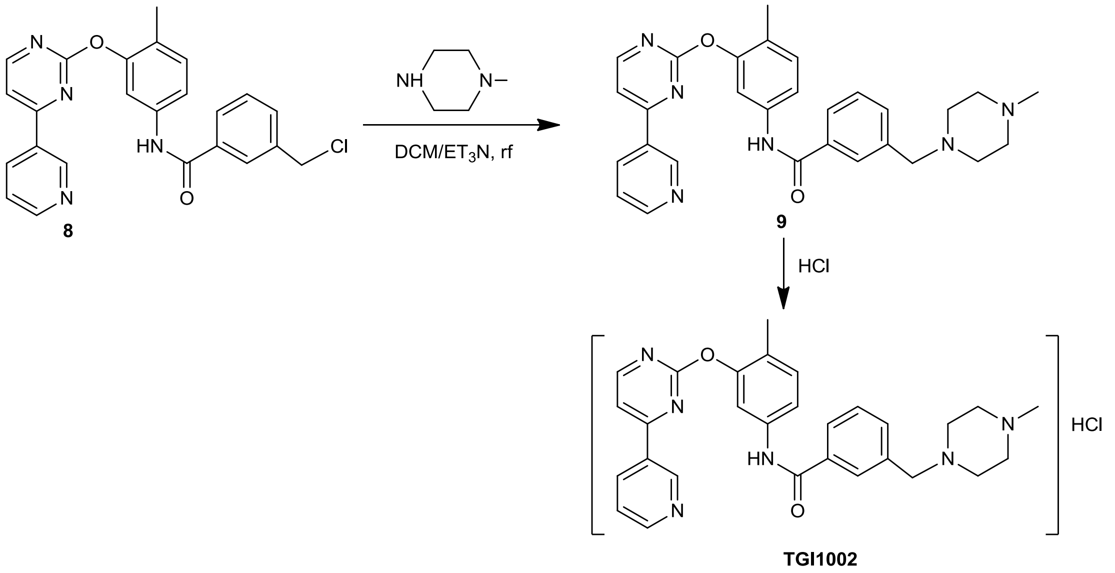

Preparation of N-(4-methyl-3-((4-(pyridin-3-yl)pyrimidin-2-yl)oxy)phenyl)-3-((4-methylpiperazin-1-yl)methyl)benzamide hydrochloride (TGI1002). Compound **8** (12.4 g, 28 mmol), 1-methylpiperazine (3.45 g, 34 mmol) and ET_3N (1.74 mL, 17 mmol) were added to 75 mL of DCM and stirred for 15 h at RT. The organic phase was collected, dried and filtered to give **9**. Compound **9** was then purified by column chromatograph (chloroform/methanol=20/1). Purified **9** (2 g) was dissolved in 5 mL methanol containing 10% HCl, and TGI1002 was then obtained as a yellow powder after removing the solvent. The total yield of TGI1002 was 60% and the purity was > 99% by HPLC analysis. Mp: 85.1-89.3℃; HRMS (*m/z*): $[M]^+$ calcd. for $C_{29}H_{30}N_6O_2$ 495.2430, observed 495.2525; ^1H NMR (300 MHz, D_2O): δ 9.39 (s, 1H), 9.14 (d, 1H, J = 8.2Hz), 9.01 (d, 1H, J = 5.6 Hz), 8.75 (d, 1H, J = 5.2 Hz), 8.23~8.28 (m, 1H), 8.05 (s, 1H), 7.84~7.95 (m, 3H), 7.67~7.72 (m,1H), 7.52 (s, 1H), 7.30 (brs, 2H), 4.68 (s, 2H), 3.84 (br, 8H), 3.17 (s, 3H), 2.09 (s, 3H); ^{13}C NMR (75 MHz, D_2O): δ 165.4, 162.4, 160.0, 159.0, 148.4, 142.7, 141.2, 138.6, 134.4, 133.2, 133.1, 132.9, 130.0, 128.5, 128.2, 127.5, 126.5, 125.9, 125.7, 117.5, 113.3, 111.6, 58.1, 48.4, 46.5, 41.1, 13.1; IR (KBr) *γ* 3408.0, 3061.2, 2927.4, 2644.3, 2562.0, 1635.8, 1582.6, 1566.9, 1505.9, 1454.9, 1385.8, 1284.9, 1263.0, 1207.7, 1095.3, 1012.5, 949.0, 812.2, 742.7, 670.6 cm^{-1}.

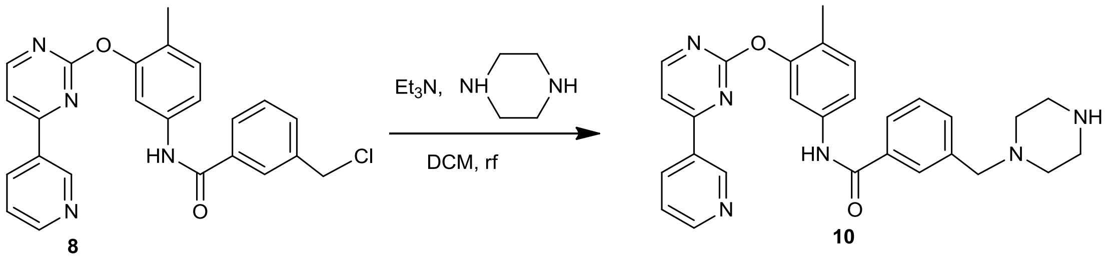

8 10

Preparation of N-(4-methyl-3-((4-(pyridin-3-yl)pyrimidin-2-yl)oxy)phenyl)-3-(piperazin-1-yl-methyl)benzamide (10). Synthesis and purification of compound **10** was performed with the same procedures as compound **9** except using piperazine to replace 1-methyl-piperazine. Mp: 86.2-90.1 ℃; HRMS (*m/z*): $[M]^+$ calcd. for $C_{28}H_{28}N_6O_2$ 481.2274, observed 481.2349; ^1H NMR (300 MHz, D_2O): δ 9.33 (s, 1H), 9.11 (d, 1H, *J* = 8.2 Hz), 8.90 (d, 1H, *J* = 5.5 Hz), 8.68 (d, 1H, *J* = 5.2 Hz), 8.16~8.20 (m, 1H), 7.95 (br, 1H), 7.90~7.94 (m, 2H), 7.82 (d, 1H, *J* = 5.2 Hz), 7.60~7.75 (m, 2H), 7.46 (s,1H), 7.32 (brs, 2H), 4.55 (s, 2H), 3.62 (br, 8H), 2.06 (s, 3H); ^{13}C NMR (75 MHz, D_2O): δ 170.6, 167.0, 165.0, 164.4, 163.8, 153.0, 147.7, 145.4, 143.1, 138.8, 137.9, 137.6, 137.5, 134.5, 133.0, 132.6, 132.0, 130.8, 130.7, 130.4, 122.6, 118.3, 116.2, 62.9, 50.7, 43.3, 17.5; IR (KBr) γ 3407.8, 2928.6, 2709.7, 2571.8, 1661.5, 1634.4, 1584.3, 1567.5, 1506.4, 1445.7, 1387.0, 1286.7, 1262.3, 1211.2, 1162.7, 1095.3, 1013.1, 951.2, 811.2, 669.7 cm^{-1}.

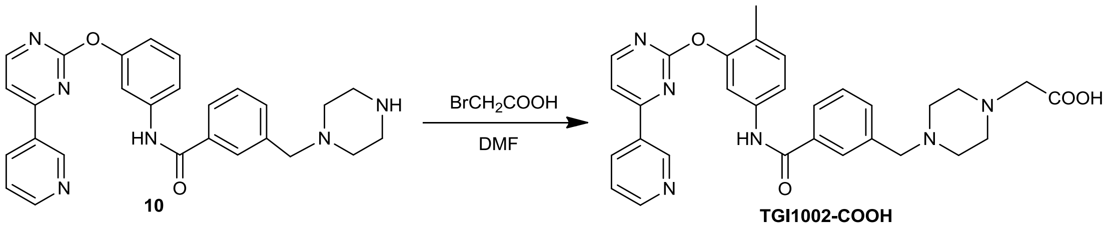

Preparation of 2-(4-(3-((4-methyl-3-((4-(pyridin-3-yl)pyrimidin-2-yl)oxy)-phenyl)carbamoyl)benzyl)piperazin-1-yl)acetic acid (TGI1002-COOH). TGI1002-COOH was synthesized from compound **10** based on previous report (1). Mp: 89.1-104.2℃; HRMS (*m/z*): $[M]^+$ calcd. for $C_{30}H_{30}N_6O_4$ 539.2329, observed 539.2440; ^{1}H NMR (300 MHz, DMSO): δ 10.37 (s, 1H), 9.51 (s, 1H), 9.08 (d, 1H, *J* = 8.7Hz), 8.90 (d, 1H, *J* = 5.1Hz), 8.79 (d, 1H, *J* = 4.8Hz), 8.32 (s, 1H), 8.15~8.19 (m, 1H), 7.94 (d, 1H, *J* = 5.4 Hz), 7.81 (br, 1H), 7.64 (s, 1H), 7.46~7.49 (m, 3H), 7.32 (d, 1H, *J* = 8.1 Hz), 5.1 (s, 2H), 3.28 (br, 4H), 3.03 (br, 2H), 2.56 (br, 4H), 2.07 (s, 3H); ^{13}C NMR (75 MHz, DMSO): δ 166.8, 165.2, 162.8, 161.2, 151.3, 147.5, 144.9, 143.0, 138.3, 138.0, 135.5, 135.2, 133.2, 131.9, 129.3, 128.9, 128.2, 127.2, 126.2, 118.8, 114.8, 114.0, 63.5, 61.7, 49.8, 43.7, 16.0; IR (KBr) *γ* 3443.7, 2961.7, 2823.0, 1590.8, 1383.1, 1352.8, 1288.3, 1206.2, 1125.8, 1099.2, 1002.4 cm^{-1}.

Reference

1. D'Alessandro PL, et al. The identification of structurally novel, selective, orally bioavailable positive modulators of mGluR2. Bioorg Med Chem Lett 2010; 20: 759-762.

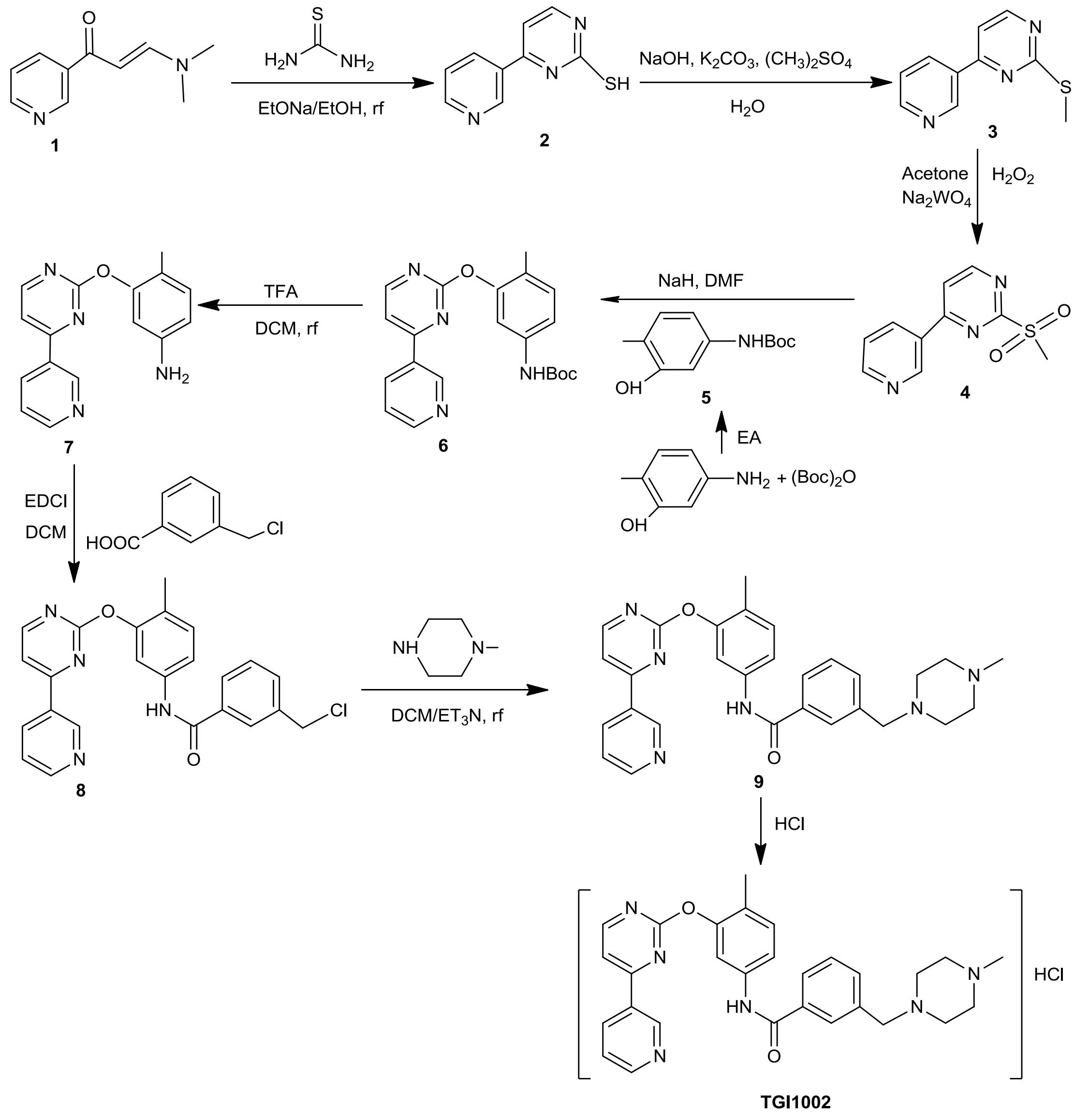

Supplementary Scheme 1: Synthetic route of TGI1002.

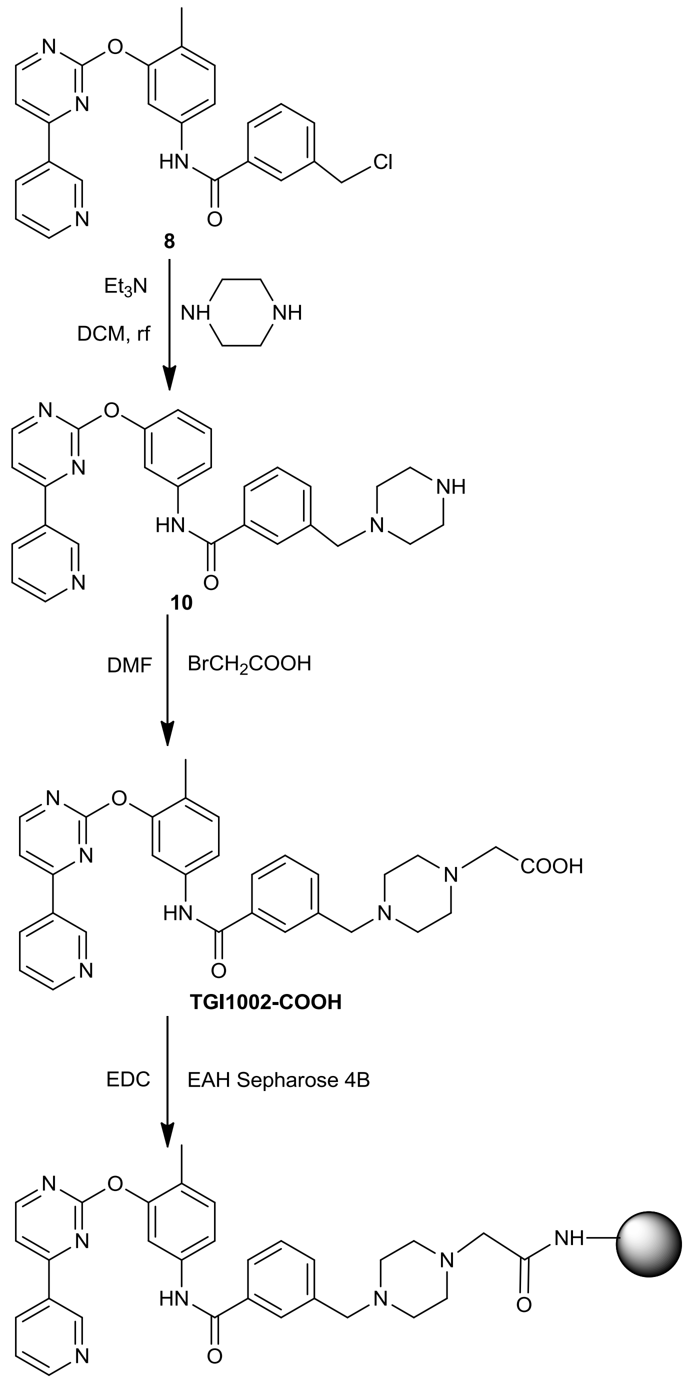

TGI1002 affinity probe

Supplementary Scheme 2: Synthesis of TGI1002 derivative as an affinity probe.

A

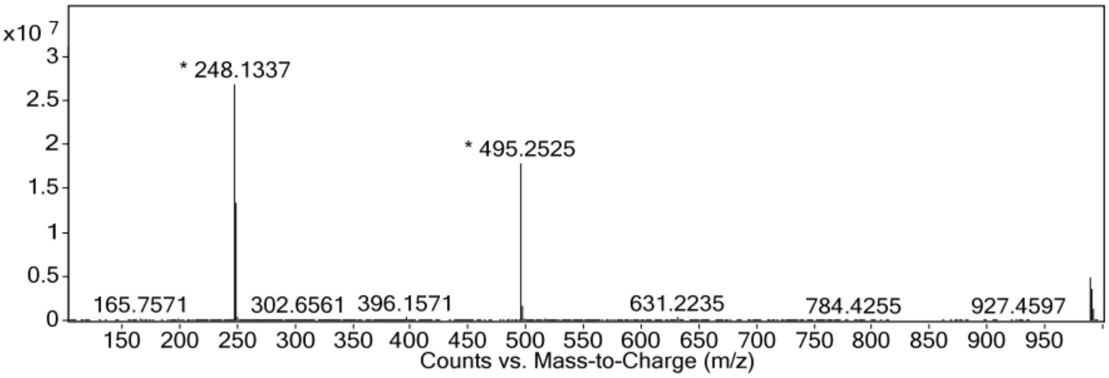
x10 7
* 248.1337
* 495.2525
165.7571
302.6561
396.1571
631.2235
784.4255
927.4597
Counts vs. Mass-to-Charge (m/z)

B

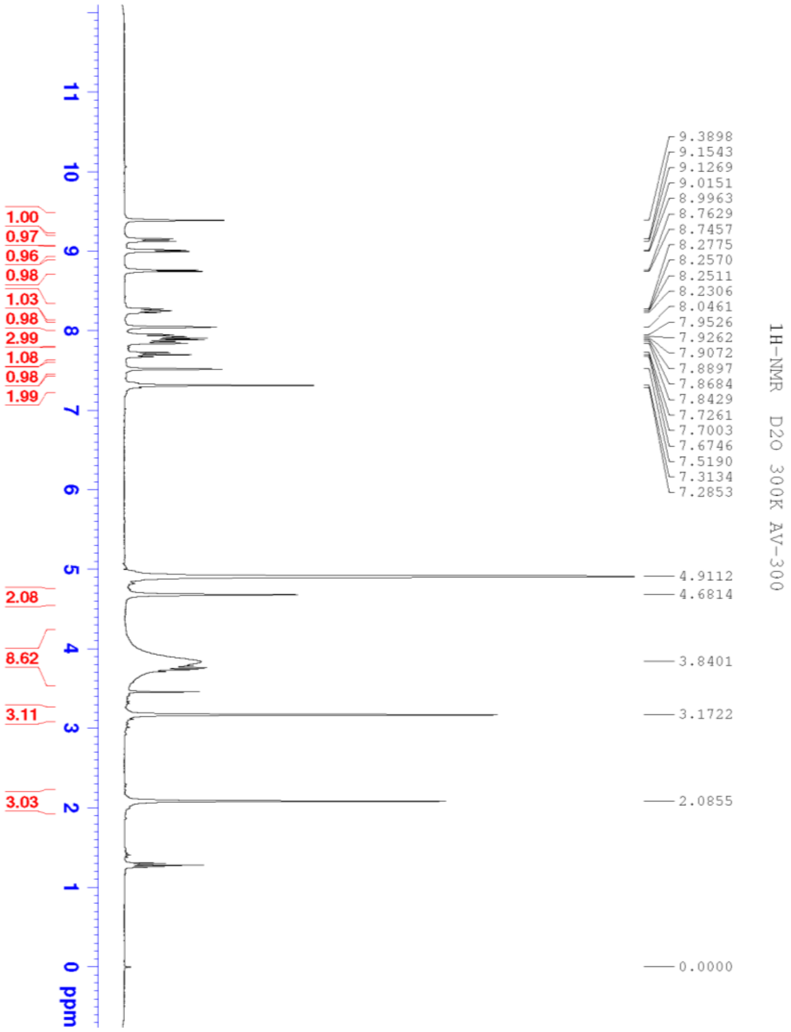
1H-NMR D2O 300K AV-300
ppm

C

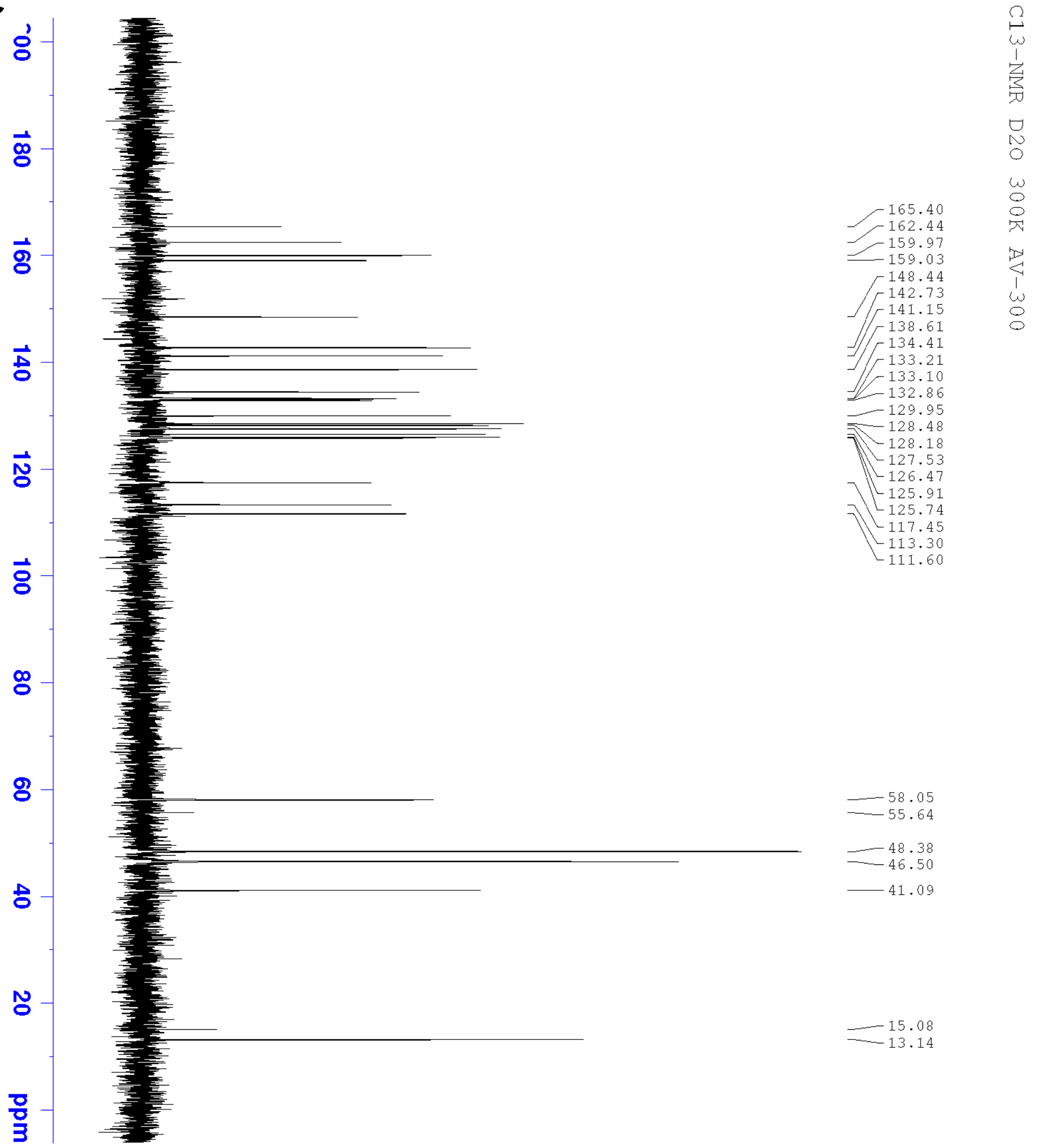
C13-NMR D2O 300K AV-300
165.40
162.44
159.97
159.03
148.44
142.73
141.15
138.61
134.41
133.21
133.10
132.86
129.95
128.48
128.18
127.53
126.47
125.91
125.74
117.45
113.30
111.60
58.05
55.64
48.38
46.50
41.09
15.08
13.14
ppm

D

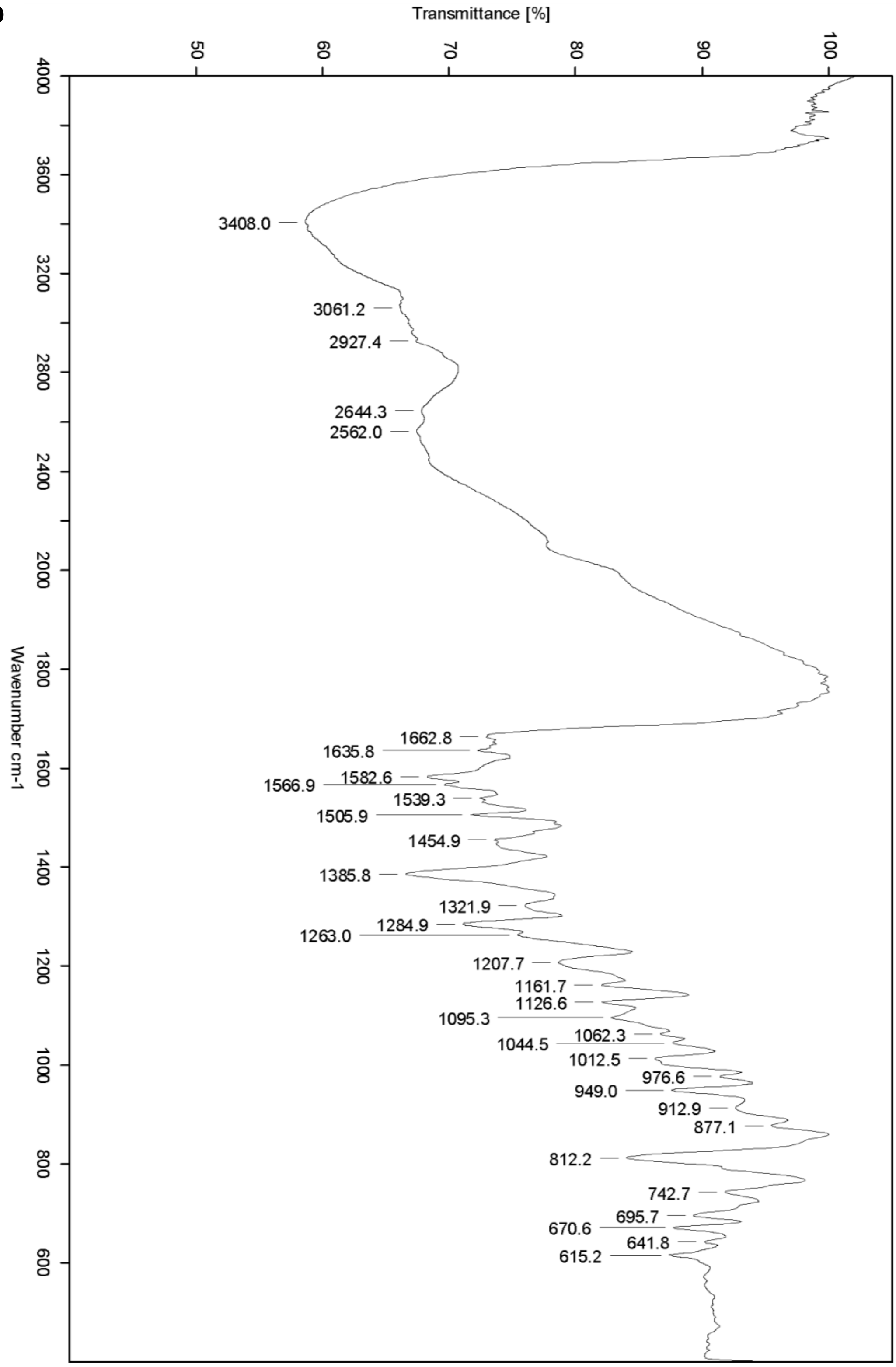

Supplementary Figure S1: HRMS, ^{1}H and ^{13}C NMR and IR analyses of TGI1002.

(**A**) Mass spectrum of TGI1002, chemical formula $C_{29}H_{30}N_6O_2$, calculated m/z

495.2430 $[M+H]^+$, observed m/z 495.2525 $[M+H]^+$. (**B**) ^1H NMR (300 MHz, D_2O). (**C**) ^{13}C NMR (75 MHz, D_2O). (**D**) IR spectrum.

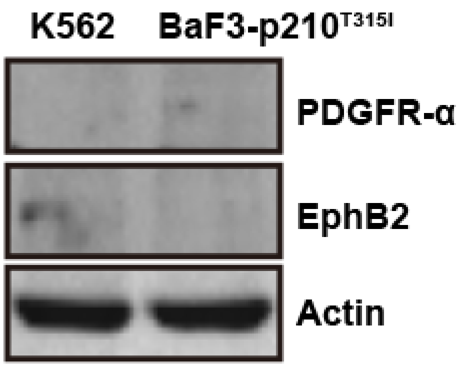

Supplementary Figure S2: Western blots of PDGFRα and EphB2 expression in K562and BaF3-p210^{T315I}cells. Cell lysates from K562 and BaF3-p210^{T315I} cells were run on SDS-PAGE and Western blotted with anti-PDGFRα, anti-EphB2 or anti-β-actin antibodies. Neither PDGFRα nor EphB2 was observed in K562 and BaF3-p210^{T315I} cell lysates.

A

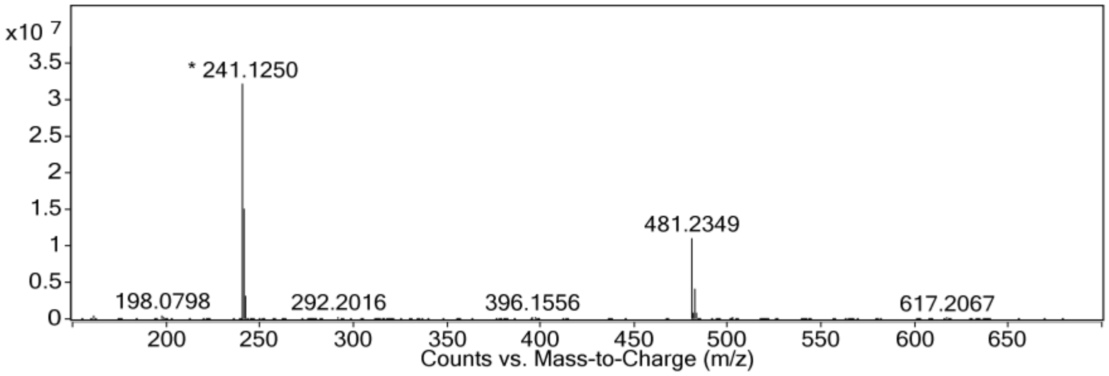
x10 7
* 241.1250
481.2349
198.0798
292.2016
396.1556
617.2067
Counts vs. Mass-to-Charge (m/z)

B

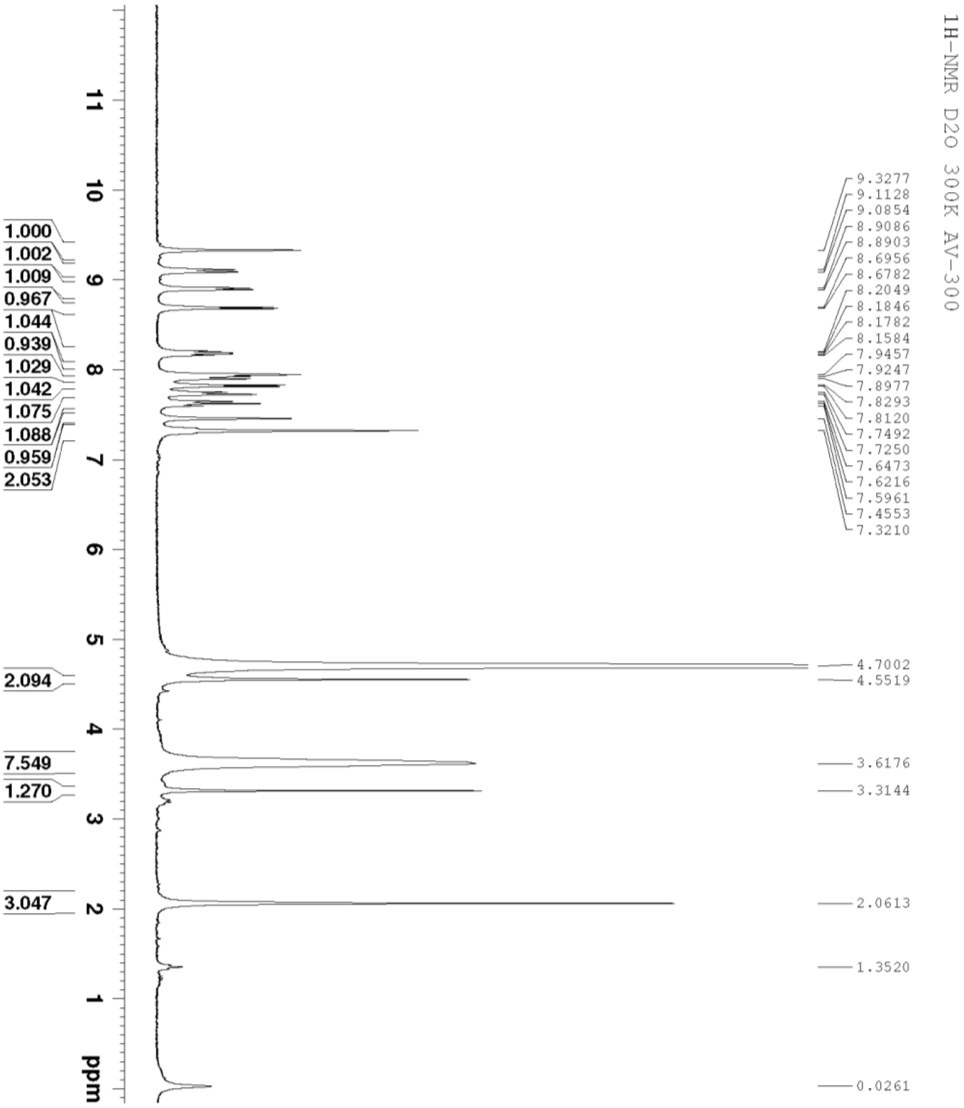
1H-NMR D2O 300K AV-300
ppm

C

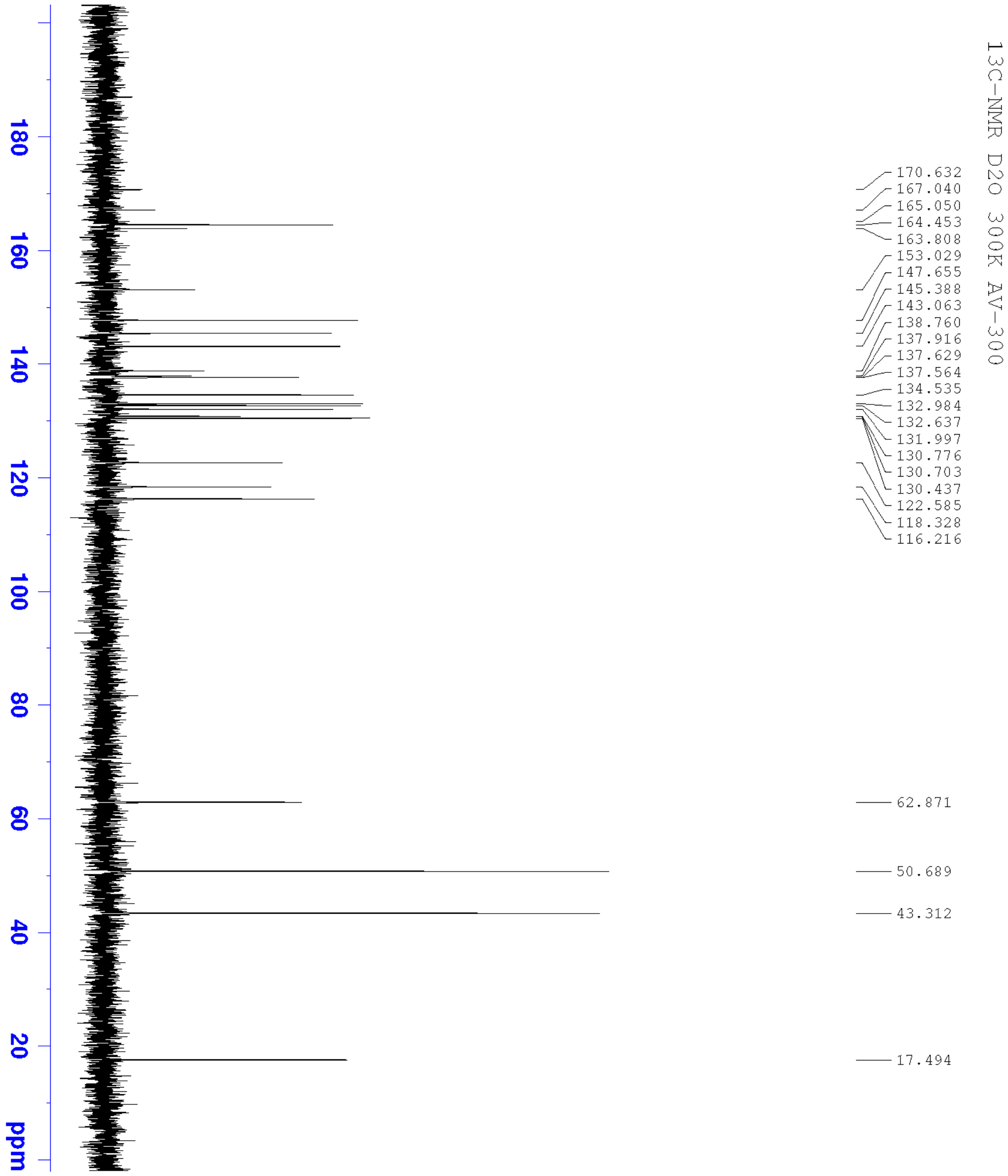
13C-NMR D2O 300K AV-300
170.632
167.040
165.050
164.453
163.808
153.029
147.655
145.388
143.063
138.760
137.916
137.629
137.564
134.535
132.984
132.637
131.997
130.776
130.703
130.437
122.585
118.328
116.216
62.871
50.689
43.312
17.494
180
160
140
120
100
80
60
40
20
ppm

D

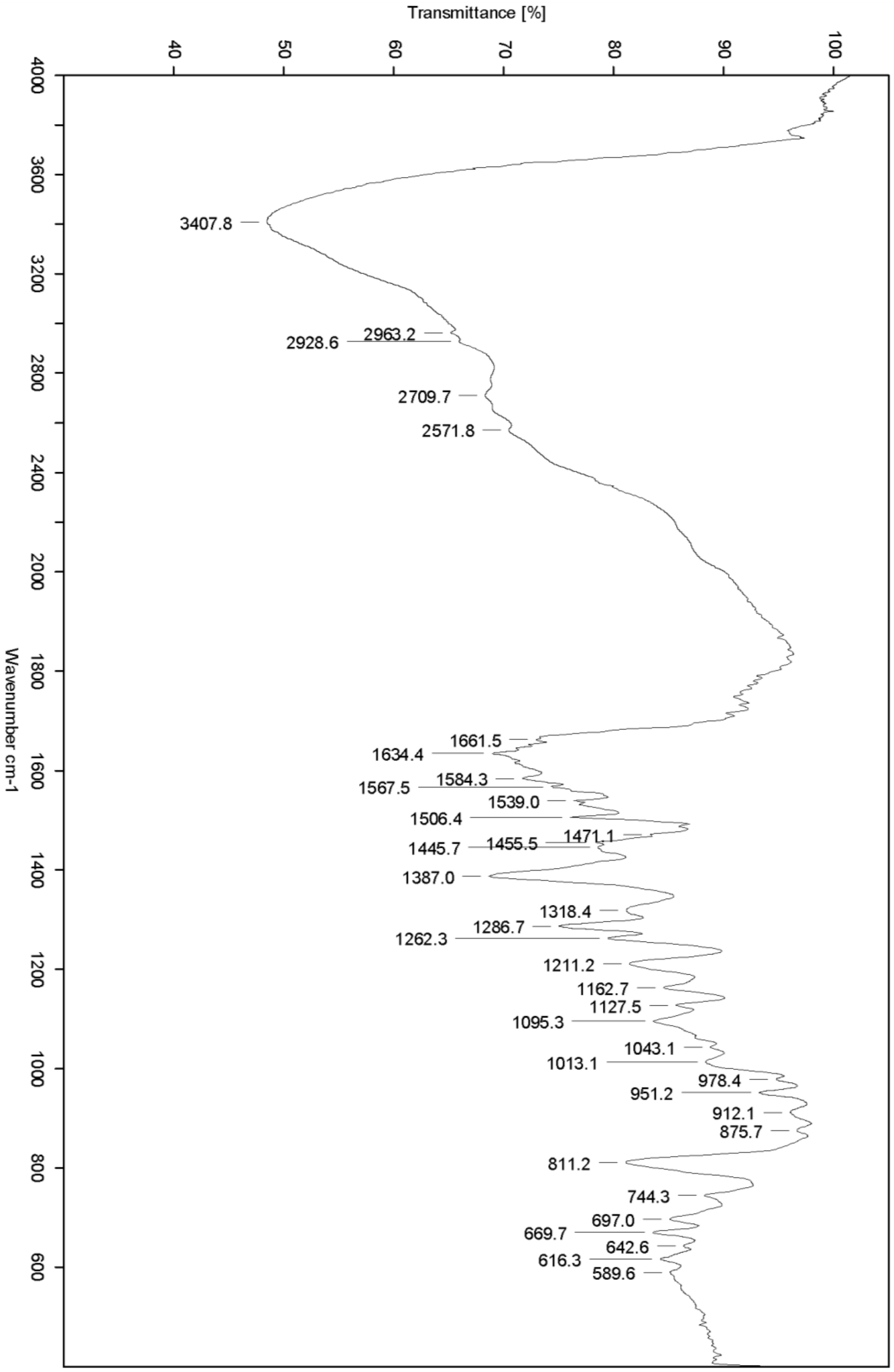

Supplementary Figure S3: HRMS, ^{1}H and ^{13}C NMR and IR analyses of 10. (**A**) Mass spectrum of compound **10**, chemical formula $C_{28}H_{28}N_6O_2$, calculated m/z

481.2274 [M+H]$^+$, observed m/z 481.2349 [M+H]$^+$. (**B**) ^{1}H NMR (300 MHz, D_2O). (**C**) ^{13}C NMR (75 MHz, D_2O). (**D**) IR.

A

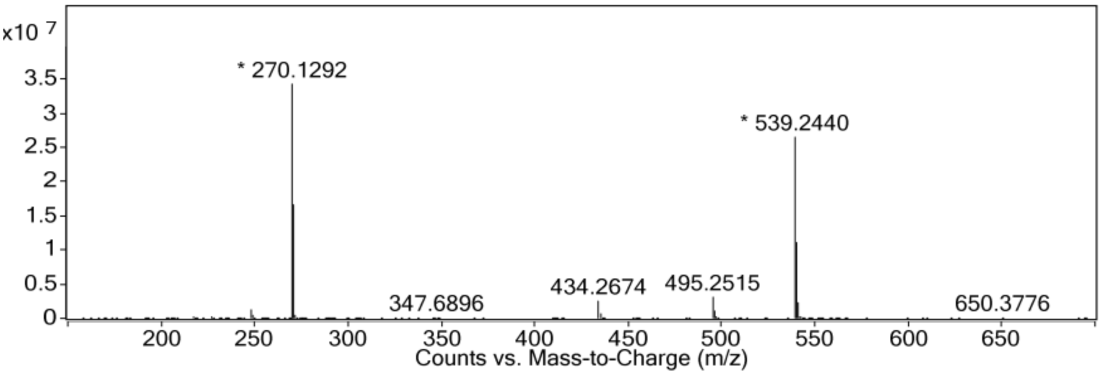

B

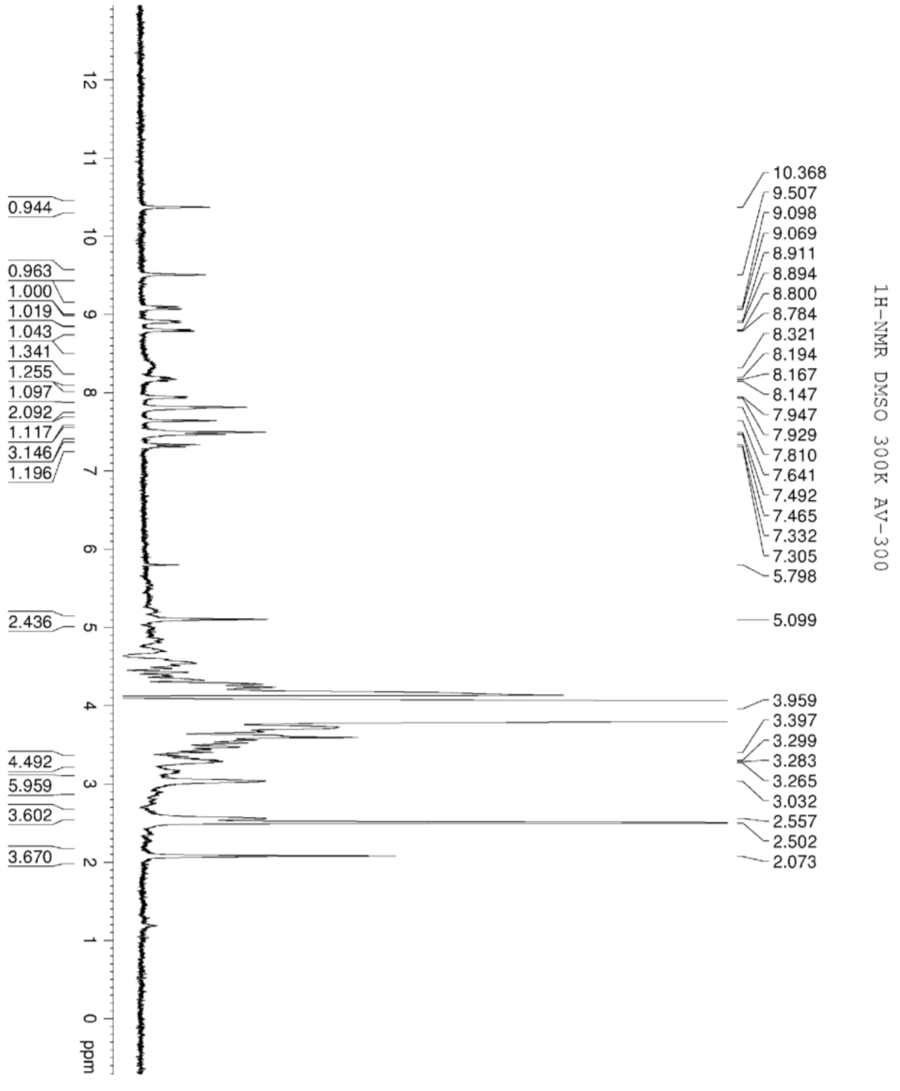

C

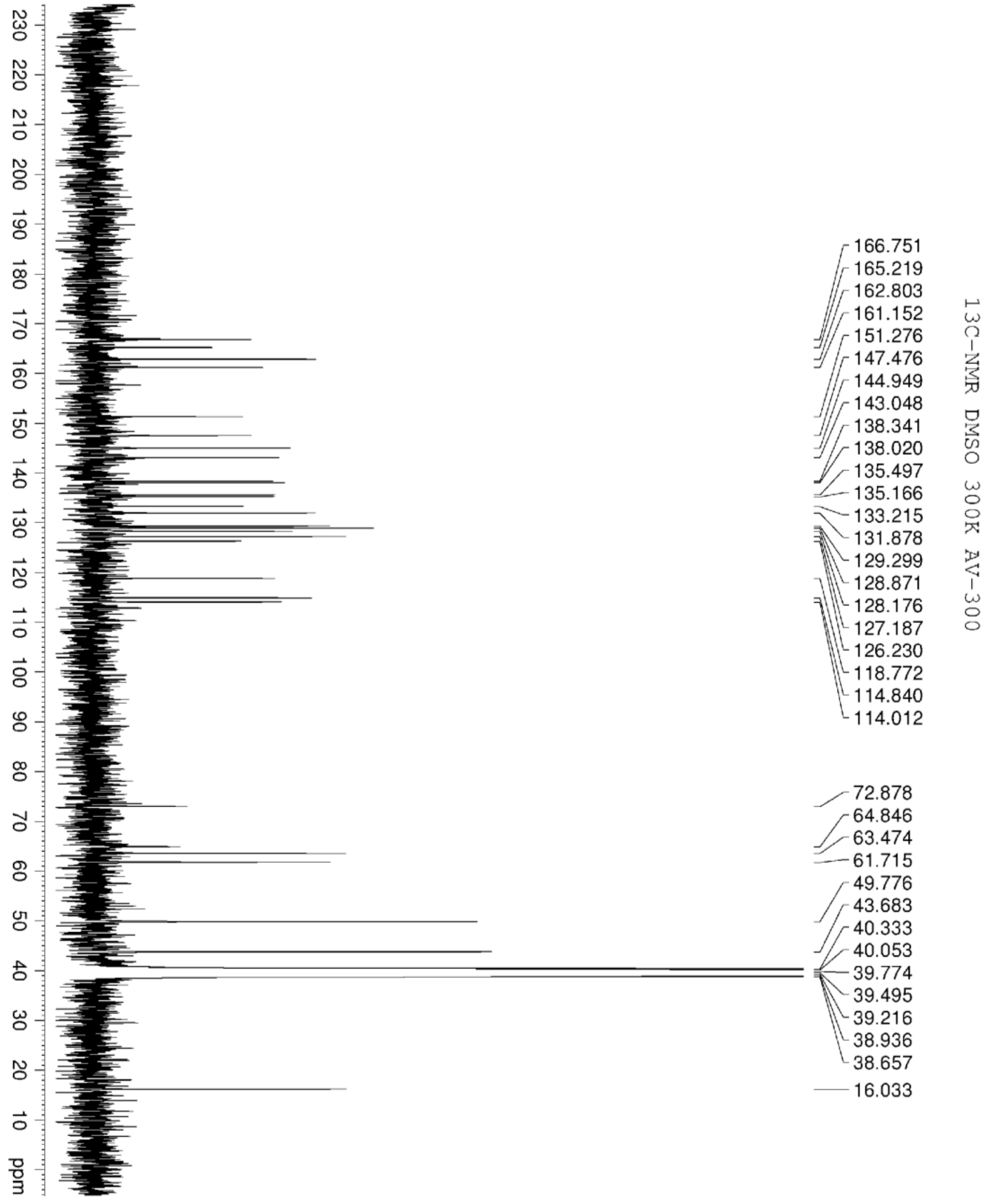
166.751
165.219
162.803
161.152
151.276
147.476
144.949
143.048
138.341
138.020
135.497
135.166
133.215
131.878
129.299
128.871
128.176
127.187
126.230
118.772
114.840
114.012
72.878
64.846
63.474
61.715
49.776
43.683
40.333
40.053
39.774
39.495
39.216
38.936
38.657
16.033
13C-NMR DMSO 300K AV-300
ppm

D

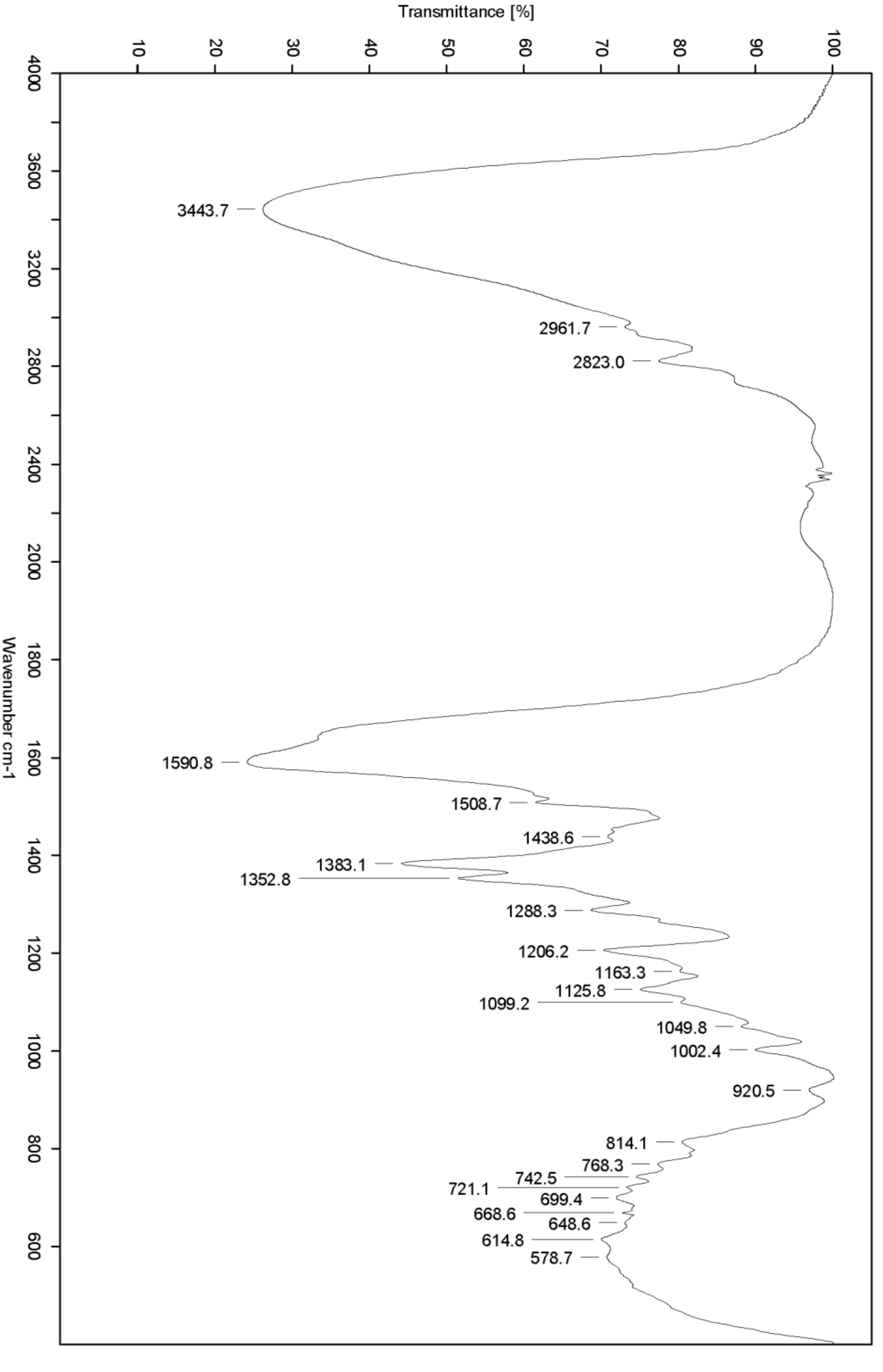

Supplementary Figure S4: HRMS, ^{1}H and ^{13}C NMR and IR analyses of TGI1002-COOH. (**A**) Mass spectrum of TGI1002-COOH, chemical formula

$C_{30}H_{30}N_6O_4$, calculated m/z 539.2329 $[M+H]^+$, observed m/z 539.2440 $[M+H]^+$. (**B**) ^1H NMR (300 MHz, DMSO). (**C**) ^{13}C NMR (75 MHz, DMSO). (**D**) IR.

A

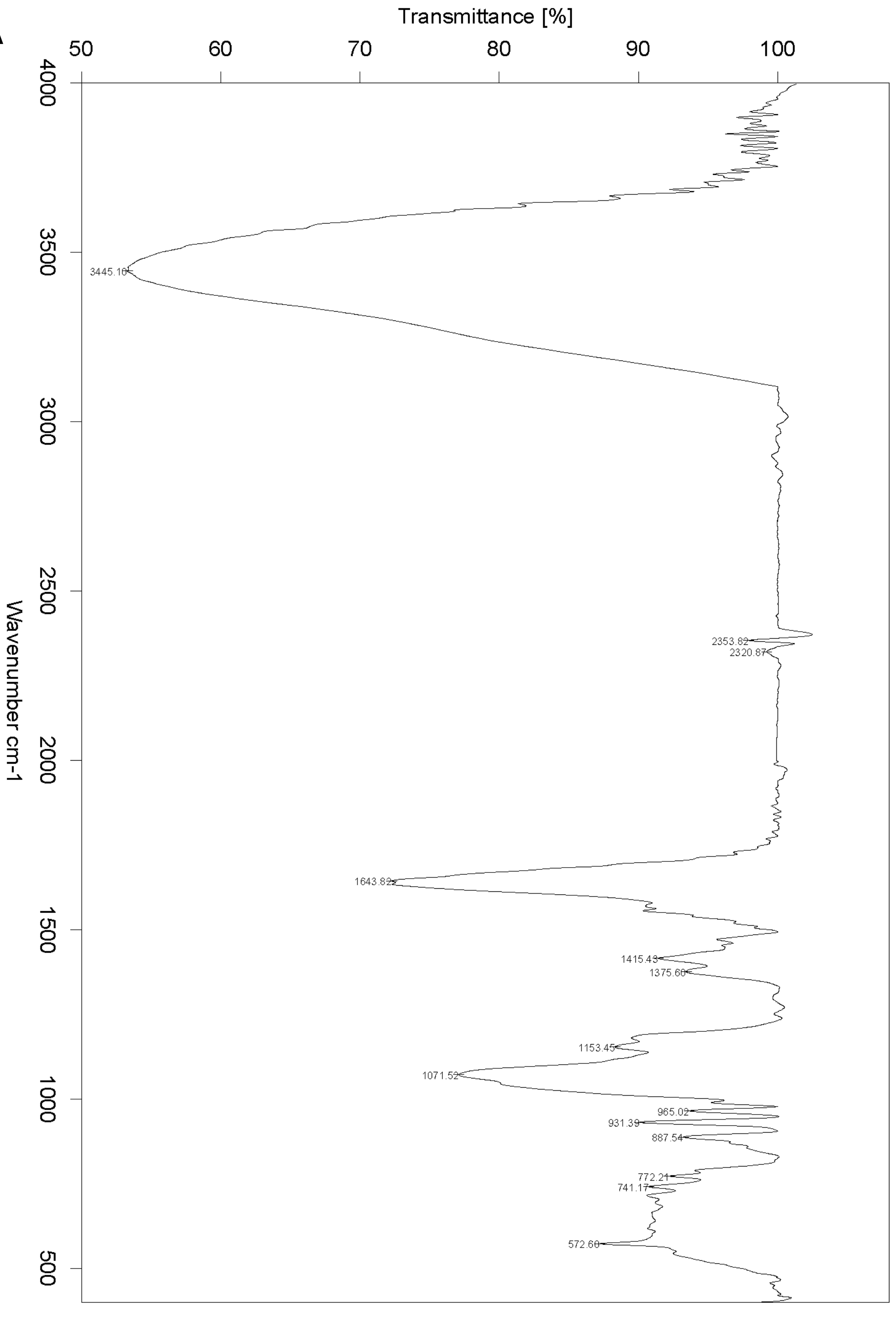
Transmittance [%]
50
60
70
80
90
100
4000
3500
3000
2500
2000
1500
1000
500
Wavenumber cm-1
3445.10
2353.82
2320.87
1643.82
1415.43
1375.66
1153.45
1071.52
965.02
931.39
887.54
772.21
741.17
572.68

B

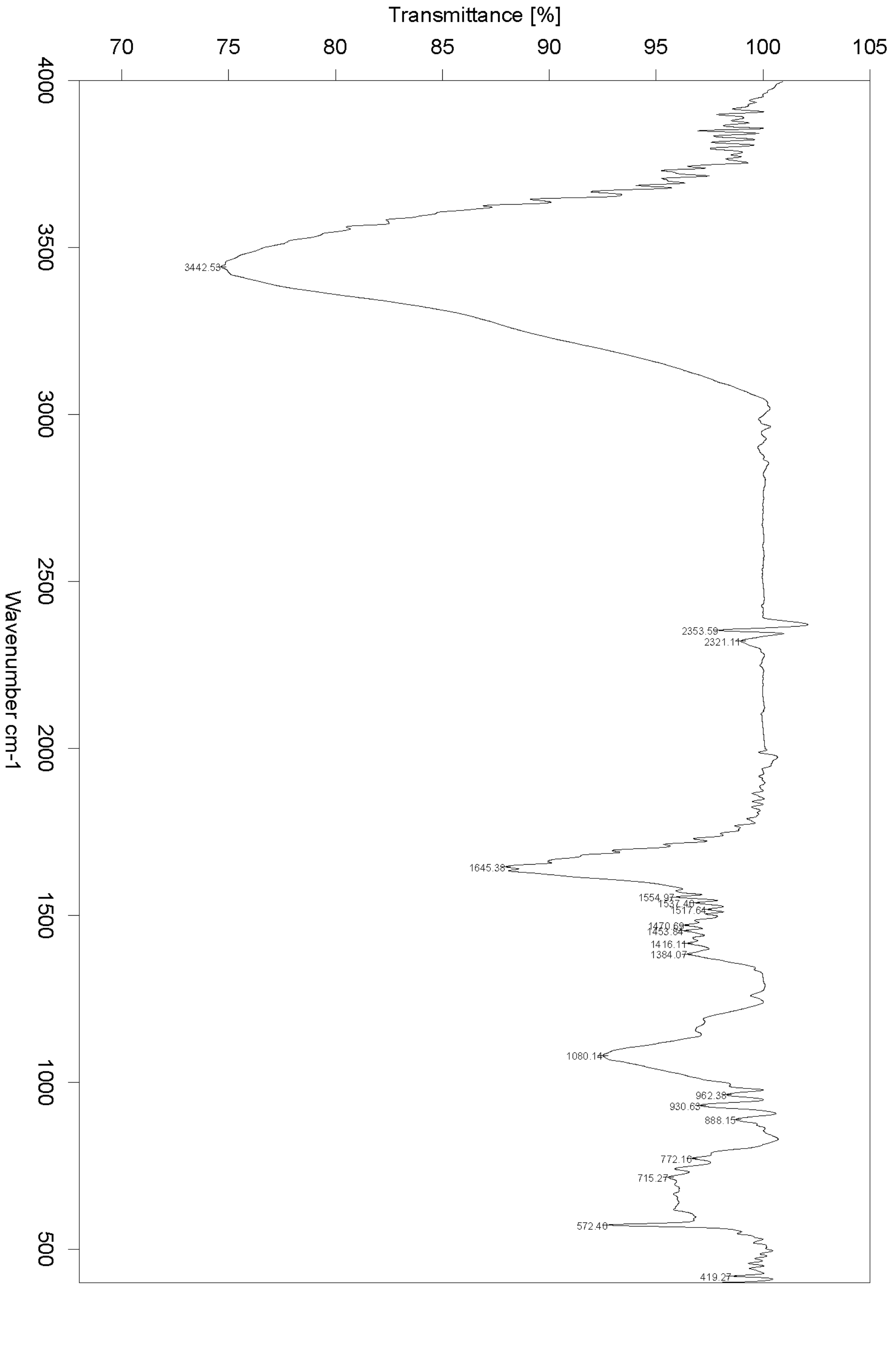

Supplementary Figure S5: IR spectral analysis of TGI1002-affinity probe. (**A**) EAH-Sepharose 4B. (**B**) TGI1002-affinity probe.

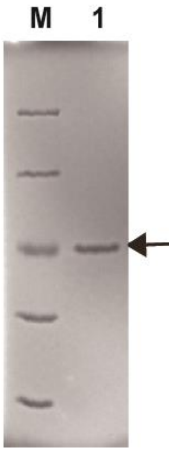

Supplementary Figure S6: SDS-PAGE of purified SET protein. *Lane M,* protein molecular weight markers. Arrow shows purified recombinant SET.

Supplementary Table S1: *In vitro* profile on kinase inhibition of TGI1002 (1 μM).

Kinase name	Inhibitory effect (%)	IC_{50} (μM)	Kinase name	Inhibitory effect (%)	IC_{50} (μM)
ABL1	4.30	N.D.	GSK3α	2.60	N.D.
ABL2	19.18	N.D.	GSK3β	2.59	N.D.
AKT1	5.07	N.D.	HER2	7.88	N.D.
AKT2	4.72	N.D.	IGF1R	8.65	N.D.
AKT3	3.62	N.D.	InsR	3.70	N.D.
ALK	4.27	N.D.	JNK1	0.20	N.D.
AMPK (A1/B1/G1)	0.19	N.D.	KDR	0.64	N.D.
AMPK (A2/B1/G1)	2.57	N.D.	LCK	5.82	N.D.
Aurora A	24.58	N.D.	NEK2	1.56	N.D.
Aurora B	0.00	N.D.	p38α	2.17	N.D.
AXL	3.03	N.D.	p38β	0.20	N.D.
BLK	3.39	N.D.	**PDGFRα**	**43.53**	**3.28**
BRAF	2.30	N.D.	PDGFRβ	17.16	N.D.
BRAF(v599E)	3.61	N.D.	PKAcα	5.96	N.D.
CAMK1	4.93	N.D.	PKAcβ	6.50	N.D.
CDK1/CyclinA2	5.27	N.D.	PKAcr	1.45	N.D.
CDK2/CyclinA2	1.54	N.D.	PKCα	5.57	N.D.
CDK1/CyclinB	3.24	N.D.	PKCγ	0.79	N.D.
CDK4/CyclinD1	7.54	N.D.	PKCξ	13.29	N.D.
CHK1	3.61	N.D.	PLK1	1.63	N.D.
c-KIT	15.25	N.D.	PLK2	3.52	N.D.
c-KIT(V654A)	11.68	N.D.	PLK3	4.71	N.D.
EGFR	0.00	N.D.	RAF1	5.37	N.D.
EGFR(T790M,L858R)	8.74	N.D.	RET	12.16	N.D.
EphA1	1.58	N.D.	RON	0.14	N.D.
**EphB2**	**46.69**	**1.27**	SRC	1.09	N.D.
ERK1	0.01	N.D.	TrkA	3.11	N.D.
ERK2(MAPK1)	0.00	N.D.	TrkB	5.58	N.D.
FGFR1	9.71	N.D.	PI3Kα	2.66	N.D.
FGR	7.53	N.D.	PI3Kβ	0.00	N.D.
FLT1(VEGFR1)	1.97	N.D.	PI3Kγ	2.65	N.D.
FLT3	2.97	N.D.	PI3Kδ	0.71	N.D.

Data represent the means of two independent experiments. N.D. = not determined.

Supplementary Table S2: MALDI-TOF-TOF analysis of TGI1002 binding proteins.

Name	NCBI ID	Peptide Matched	Sequence Coverage (%)	Theroetical Mr/pI	Protein Name
BP1	gi|12654329	22	46	64749/5.1	HSP90AA1 protein
BP2	gi|338695	20	53	50240/4.75	Beta-tubulin
BP3	gi|512485	14	44	47498/8.39	Acetylcholine receptor-associated protein (Rapsyn)
BP4	gi|145843637	7	35	26593/4.73	SET protein
